# Polycystic ovary syndrome, androgen excess, and the risk of nonalcoholic fatty liver disease in women: A longitudinal study based on a United Kingdom primary care database

**DOI:** 10.1371/journal.pmed.1002542

**Published:** 2018-03-28

**Authors:** Balachandran Kumarendran, Michael W. O’Reilly, Konstantinos N. Manolopoulos, Konstantinos A. Toulis, Krishna M. Gokhale, Alice J. Sitch, Chandrika N. Wijeyaratne, Arri Coomarasamy, Wiebke Arlt, Krishnarajah Nirantharakumar

**Affiliations:** 1 Institute of Applied Health Research, University of Birmingham, Birmingham, United Kingdom; 2 Department of Public Health, Faculty of Medicine, University of Kelaniya, Kelaniya, Sri Lanka; 3 Institute of Metabolism and Systems Research, University of Birmingham, Birmingham, United Kingdom; 4 Centre for Endocrinology, Diabetes and Metabolism, Birmingham Health Partners, Birmingham, United Kingdom; 5 Department of Obstetrics and Gynaecology, Faculty of Medicine, University of Colombo, Colombo, Sri Lanka; University of Manchester, UNITED KINGDOM

## Abstract

**Background:**

Androgen excess is a defining feature of polycystic ovary syndrome (PCOS), which affects 10% of women and represents a lifelong metabolic disorder, with increased risk of type 2 diabetes, hypertension, and cardiovascular events. Previous studies have suggested an increased risk of nonalcoholic fatty liver disease (NAFLD) in individuals with PCOS and implicated androgen excess as a potential driver.

**Methods and findings:**

We carried out a retrospective longitudinal cohort study utilizing a large primary care database in the United Kingdom, evaluating NAFLD rates in 63,120 women with PCOS and 121,064 age-, body mass index (BMI)-, and location-matched control women registered from January 2000 to May 2016. In 2 independent cohorts, we also determined the rate of NAFLD in women with a measurement of serum testosterone (*n* = 71,061) and sex hormone-binding globulin (SHBG; *n* = 49,625). We used multivariate Cox models to estimate the hazard ratio (HR) for NAFLD and found that women with PCOS had an increased rate of NAFLD (HR = 2.23, 95% CI 1.86–2.66, *p* < 0.001), also after adjusting for BMI or dysglycemia. Serum testosterone >3.0 nmol/L was associated with an increase in NAFLD (HR = 2.30, 95% CI 1.16–4.53, *p* = 0.017 for 3–3.49 nmol/L and HR = 2.40, 95% CI 1.24–4.66, *p* = 0.009 for >3.5 nmol/L). Mirroring this finding, SHBG <30 nmol/L was associated with increased NAFLD hazard (HR = 4.75, 95% CI 2.44–9.25, *p* < 0.001 for 20–29.99 nmol/L and HR = 4.98, 95% CI 2.45–10.11, *p* < 0.001 for <20 nmol/L). Limitations of this study include its retrospective nature, absence of detailed information on criteria used to diagnosis PCOS and NAFLD, and absence of data on laboratory assays used to measure serum androgens.

**Conclusions:**

We found that women with PCOS have an increased rate of NAFLD. In addition to increased BMI and dysglycemia, androgen excess contributes to the development of NAFLD in women with PCOS. In women with PCOS-related androgen excess, systematic NAFLD screening should be considered.

## Abstract 2

Krishnarajah Nirantharakumar and colleagues indicate a link between high levels of androgen and the development of nonalcoholic fatty liver disease in women with polycystic ovary syndrome.

## Author summary

**Why was this study done?:**

- Polycystic ovary syndrome (PCOS) affects 10% of women and is defined by androgen excess, chronic anovulation, and polycystic appearance of the ovaries on ultrasound.
- PCOS is not only a reproductive but also a lifelong metabolic disorder with increased rates of type 2 diabetes, hypertension, and cardiovascular events.
- Recent research has shown rates of nonalcoholic fatty liver disease (NAFLD) may be higher in women with PCOS and that androgens may play a causative role in its pathogenesis.

**What did the researchers do and find?:**

- We performed a population-based retrospective cohort study utilizing a large UK primary care database and included more than 63,000 women with PCOS and 121,000 matched controls registered between 2000 and 2016.
- We found that rates of NAFLD were increased in women with PCOS.
- We found that even normal-weight women with PCOS had an increased rate of NAFLD.
- In addition to body mass index and dysglycemia, we identified androgen excess as a potential additional contributing risk factor for NAFLD development in PCOS.
- We also studied 2 independent cohorts drawn from the same primary care database who had serum testosterone (*n* = 71,000) or sex hormone-binding globulin (SHBG, *n* = 49,000) measured and found that women with biochemical evidence of androgen excess (high testosterone, low SHBG) had an increased rate of NAFLD.

**What do these findings mean?:**

- Our findings indicate there may be an increased risk of NAFLD in women with PCOS and that androgen excess is a factor underlying that risk.
- Screening for NAFLD should be considered in women with PCOS-related androgen excess.
- Future studies will need to determine if androgen excess also drives progression to liver inflammation and fibrosis and to establish whether antiandrogen medication can reduce the risk of NAFLD.

## Introduction

Polycystic ovary syndrome (PCOS) is the most common endocrine disorder in women of reproductive age, affecting 6%–10% of women worldwide [1]. Diagnostic criteria include the presence of androgen excess, oligomenorrhea, and evidence of polycystic ovaries (PCO) on ultrasound [2]. Though conventionally perceived as a reproductive disorder, PCOS is now emerging as a lifelong metabolic disorder, with evidence of increased prevalence of obesity, insulin resistance, and metabolic syndrome [3]. However, the metabolic disease burden in patients with PCOS exceeds that observed in simple obesity [4]. Androgen excess has been implicated as a distinct risk factor, with several studies showing circulating androgen burden to correlate closely with surrogate markers of metabolic risk, independent of body mass index (BMI) [5–7].

Nonalcoholic fatty liver disease (NAFLD) is a hepatic complication of the metabolic syndrome, with global NAFLD prevalence rising to epidemic proportions in recent years [8]. It encompasses a continuous spectrum of morphological changes in the liver, from simple hepatic steatosis, continuing to the stage of nonalcoholic steatohepatitis (NASH), with prevailing inflammation, and eventually progressing to irreversible hepatic fibrosis and cirrhosis [9]. Overall, patients with NAFLD have increased mortality due to an increased risk of cardiometabolic complications and death from liver failure and hepatocellular carcinoma [10]. NAFLD is now the second most prevalent indication for liver transplantation in the United States [11]. There is a clear association between NAFLD risk, insulin resistance and obesity. Adipose tissue is thought to be the principal contributor of free fatty acids and systemic lipotoxicity underpinning the development of hepatic steatosis [12].

Given the shared risk factors between PCOS and NAFLD, there is a strong interest in exploring the relationship between the 2 conditions, both in terms of prevalence and shared pathophysiological mechanisms. A systematic review of 7 studies, involving mostly small cohorts with less than 60 patients, reported an estimated 4-fold increased risk of NAFLD among patients with PCOS compared to controls [13]. Similarly, 2 small cross-sectional studies found significant associations between NAFLD and PCOS [14,15]. Following the publication of some preliminary evidence [16], 2 recent studies and a very recent meta-analysis have implicated androgen excess as a potentially BMI-independent risk factor for NAFLD in PCOS [17–19].

Our study aimed to comprehensively investigate the excess incidence of NAFLD in patients with PCOS, assess if the excess risk was independent of their BMI status, and explore the potential role of androgen excess as an independent risk factor in the development of NAFLD, by undertaking a longitudinal study drawing from a large and diverse population base.

## Methods

The study followed the preanalysis study plan (S1 Text) and is reported as per Strengthening the Reporting of Observational Studies in Epidemiology (STROBE) guidelines (S2 Text).

### Study design

This is a population-based retrospective cohort study to determine the association between PCOS (exposure) and NAFLD (outcome) and to assess predictors of NAFLD within the PCOS group. Independent cohorts were separately created to explore the association of biomarkers of androgen excess (serum testosterone and sex hormone-binding globulin [SHBG]) with NAFLD risk (S2 Fig).

### Setting

Data for this study were derived from The Health Improvement Network (THIN) database, a UK general practice electronic database. THIN data are generated from longitudinal data documented in electronic medical records by general practitioners for clinical and management purposes during each episode of consultations using Read codes, a hierarchical coding system for structured storage of information [20]. More than 675 practices, scattered representatively around UK, contribute data to THIN, covering 3.7 million active patients (6%–7% of UK population) [21]. THIN data are generalizable for the UK for major health conditions [22] and have been used for studies involving women with PCOS [23] and patients with NAFLD [24].

### Participants

Women aged 18 to 50 years at study entry were eligible to take part in the study. Patients with any documentation of excess alcohol use were excluded. Excess alcohol use was determined by Read codes indicative of hazardous drinking or diseases related to hazardous drinking (e.g., alcoholic liver disease). The exposed group comprised eligible women with a diagnosis of PCOS at any time during the study period. The nonexposed group comprised eligible women who did not have a documented diagnosis of PCOS and were matched within the same practice for age (±1 year) and BMI (±2 kg/m^2^) of each woman with PCOS. Age and BMI were used as matching variables because of their strong association with PCOS and NAFLD [25,26]. Where there were several patients who could be matched, for each exposed patient 2 were randomly selected.

### Study period

Our study period extended from 1 January 2000 (study start date) to 15 May 2016 (study end date). The follow-up start date for an indexed patient (index date) in the exposed group was set at first documentation of PCOS once a patient was eligible to take part in the study (newly diagnosed patients) or set as the date a patient became eligible to take part in the study, if they already had a diagnosis of PCOS (patients with an existing diagnosis) (S1 Fig). The same index date was assigned for their matched corresponding unexposed patients to mitigate immortality time bias [27]. The earliest of transfer date (when patient left the practice), death date, first documentation of outcome—i.e., NAFLD—(outcome date), or study end date were considered as the follow-up end date (exit date).

### PCOS definition

High similarity between “PCOS” and “polycystic ovaries (PCO)” increases the possibility of misclassification between these 2 during data entry. However, prevalence studies based on primary care data suggest that both combined together reflect true prevalence [23]. Therefore, primary analysis included both, while sensitivity analysis was carried out with codes reflecting PCOS only.

Though limitations exist in documentation of the 3 features (ovulatory dysfunction, androgen excess, and PCO) used in the diagnosis of PCOS, these were captured where available. We considered clinical androgen excess as present if clinical features commonly accepted as indicative of clinically relevant androgen excess in women (hirsutism, alopecia, or acne) were documented.

### Outcome

Our primary outcome was incidence of NAFLD.

### Independent cohorts to explore the role of androgen excess in the development of NAFLD

Two cohorts independent of the primary PCOS cohort were constructed to test the hypothesis that androgen excess has a role in women developing NAFLD. The first cohort comprised any woman with a serum testosterone measurement, and the second one comprised any woman with a serum SHBG measurement. This was essential as androgen excess features (acne, hirsutism, and hair loss) and biomarkers of androgen excess were not sufficiently documented within the PCOS cohort and therefore did not have adequate power to detect an association (S1 and S2 Tables). Both cohorts had similar entry and exit requirements as the primary cohort study (S1 Fig).

### Selection of read codes and drug codes for the cohort exposure, outcome, and covariates

Variables for PCOS, NAFLD, and covariates were defined using published methodologies [28], definitions noted in previous publications [23,24], and our own systematic search process for codes (S3 and S4 Tables). PCOS was defined using Read codes for polycystic ovary syndrome [23] and polycystic ovaries [29]. The outcome variable NAFLD was defined using nonalcoholic fatty liver and nonalcoholic steatohepatitis Read codes [24]. Other potential confounders and covariates were informed by previous literature and included the Townsend index of deprivation [30,31], diabetes mellitus or impaired glucose regulation (IGR, includes impaired fasting glucose [IFG; defined as fasting plasma glucose 6.1–6.9 mmol/L] and impaired glucose tolerance [IGT; defined as plasma glucose 7.8–11.1 mmol/L measured 120 minutes after ingestion of 75 g glucose in the oral glucose tolerance test]), hypothyroidism, metformin use, and lipid-lowering medications [23,32]. Predictor variables were also selected for analysis restricted to women with PCOS only. These included Read codes for hirsutism, acne, and alopecia and medication codes for cyproterone acetate, drospirenone, and other antiandrogen medications.

### Grouping of quantitative variables

BMI (in kg/m^2^) was categorized into <25, 25 to 30, and >30 kg/m^2^ as per the WHO recommendation for obesity measurement (normal weight, overweight, and obese).

### Statistical methods

The basic characteristics of participants, including covariates, were summarized by exposure status using appropriate summary measures. No statistical significance testing was performed to show any difference between the patients with PCOS and the control patients at baseline, in line with good practice [33,34].

#### Primary analysis and sensitivity analysis

Crude hazard ratios (HRs) were estimated, followed by adjusted estimates using Cox-regression models. We reported the HR estimates with 95% confidence intervals. Covariates in Cox models for main analysis included age, BMI category, Townsend category, hypothyroidism, and diabetes mellitus status. Diabetes status at baseline and follow-up were both used in separate analyses. Analyses were undertaken using diabetes status during follow-up as a covariate to assess the impact of PCOS on the development of NAFLD not associated with the development of diabetes. Analyses were also undertaken by excluding patients with diabetes and IGR at baseline and by censoring when they developed diabetes or IGR subsequently during follow-up. This enabled us to assess if the hazard of developing NAFLD was independent of the subsequent development of diabetes or IGR.

Sensitivity analysis was performed to assess the possibility of bias due to case definition (PCOS and PCO versus PCOS codes only) and survival bias due to including prevalent cases with PCOS (diagnosed with PCOS before meeting eligibility criteria) in the analysis [35].

#### Analysis to assess if NALFD risk in women with PCOS was independent of BMI

First, we conducted a subgroup analysis within each BMI category to assess if the NALFD risk was independent of BMI status. In addition, interaction terms were used to assess possible interaction between the exposure (PCOS/PCO) and BMI categories and their combined effect on NAFLD risk.

#### Analysis to explore if androgen excess in women with PCOS confers a risk for NALFD

Firstly, we introduced the clinical androgen excess features into the primary model to see if it attenuated the effect size. A subsequent analysis limited to exposed patients was carried out to identify predictors of NAFLD in women with PCOS with phenotypes and antiandrogenic medications as covariates in addition to previously stated covariates. These 2 analyses were limited given that only a third of patients had their androgen excess features documented.

Finally, we carried out an analysis of the 2 independent cohorts (women with a serum testosterone measurement or an SHBG measurement, respectively). Serum SHBG and testosterone categories were derived based on clinically meaningful cutoff values and by consensus among the study group to demonstrate any dose response relationship. Serum SHBG concentrations were categorized into <20, 20–29.99, 30–39.99, 40–40.99, 50–59.99, and ≥60 nmol/L, and serum testosterone concentrations were categorized into <1, 1–1.49, 1.5–1.99, 2–2.49, 2.5–2.99, 3–3.49, and ≥3.5 nmol/L. Association between these hormonal levels and NAFLD was assessed using Cox regression models adjusting for potential covariates. All data cleaning and analysis were carried out using STATA MP version 14.2.

### Ethical approval

The use of the THIN data for research was approved by the South-East Multicenter Research Ethics Committee in 2003 [36] without the need for informed consent. As per requirement of the ethical approval, further registration and authorization for this project were obtained from the relevant Scientific Review Committee (17THIN026).

## Results

### Study population characteristics

**Fig 1:**
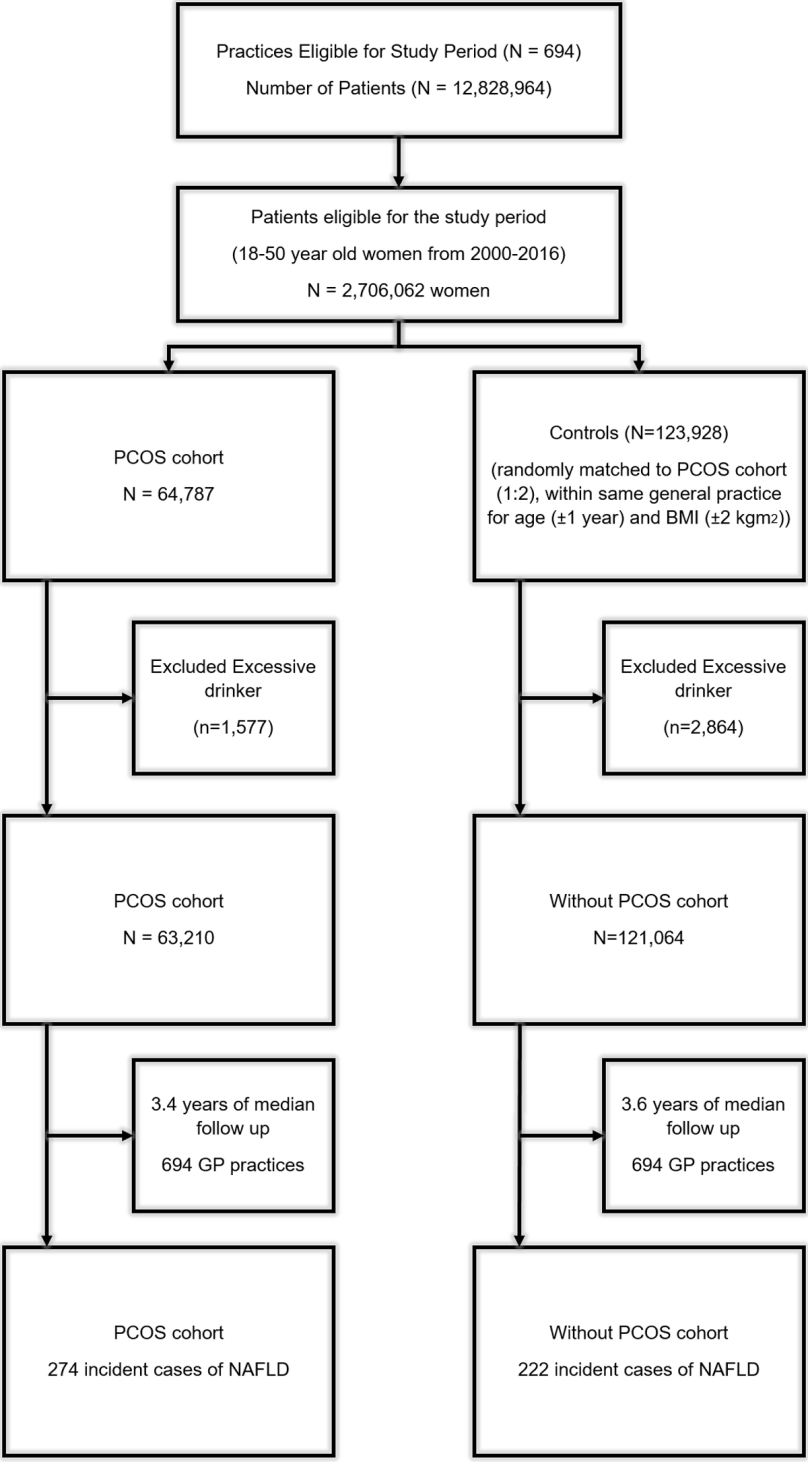
Selection of patients with polycystic ovary syndrome (PCOS) and controls. A total of 694 general practices were eligible for inclusion in the study, with a total of 2,706,062 women for the study period 2000–2016. A total of 64,787 women were listed as having PCOS according to the criteria defined in the “Methods” section. Controls were randomly matched to women with PCOS at a ratio of 2:1 within each practice, generating a random control cohort of 123,928 women. Participants with a documented history of alcohol excess were excluded; therefore, the final cohort consisted of 63,120 women with PCOS and 121,064 controls.

The study population included 63,210 women with PCOS and 121,064 women without PCOS, matched for age, BMI, and general practice (Fig 1). Both cohorts were followed up for a median of 3.5 years (IQR 1.4–7.1 years). The median age of participants was 30 years (IQR 25.2–35.4 years), with no apparent imbalance in distribution of age, Townsend index, and smoking status between groups. At baseline, women with PCOS were more likely to have diabetes (2.2% versus 1.4% in controls), hypertension (3.1% versus 2.4%), and hypothyroidism (3.9% versus 2.5%) (Table 1). During follow-up, 2.1% of women in the PCOS cohort developed diabetes in comparison to 1.1% of women in the unexposed cohort.

**Table 1:** Baseline characteristics of the 63,210 women with polycystic ovary syndrome (PCOS) and 121,064 control women without PCOS.

Characteristics	PCOS	Controls
**Age** mean (SD)	30.6 (7.1)	30.8 (7.1)
**Townsend score**^**#**^ *n* (%)		
1	12,410 (19.6)	23,540 (19.4)
2	11,224 (17.8)	21,126 (17.4)
3	13,238 (20.9)	25,629 (21.3)
4	12,634 (20.0)	24,930 (20.6)
5	8,737 (13.8)	17,596 (14.5)
Missing data	4,967 (7.9)	8,243 (6.8)
**BMI (kg/m**^**2**^**) categories** *n* (%)		
<25	21,689 (34.3)	44,742 (37.0)
25–30	12,412 (19.6)	24,656 (20.4)
>30	19,499 (30.8)	32,627 (27.0)
Missing or implausible data	9,610 (15.2)	19,039 (15.7)
**Smoking status** *n* (%)		
Nonsmokers	46,133 (73.0)	88,519 (73.1)
Smokers	14,191 (22.4)	29,272 (24.2)
Missing or implausible data	2,886 4.6)	3,273 2.7)
**Medical conditions at baseline** *n* (%)		
Diabetes	1,378 (2.2)	1,693 (1.4)
Hypertension	1,953 (3.1)	2,839 (2.4)
Hypothyroidism	2,462 (3.9)	3,009 (2.5)
IGR*	348 (0.6)	384 (0.3)

^#^Townsend score of social deprivation: presented as quintiles with 1 as the least deprived and 5 as the most deprived.

*IGR, impaired glucose regulation (includes impaired fasting glucose [IFG; defined as fasting plasma glucose 6.1–6.9 mmol/L] and impaired glucose tolerance [IGT; defined as plasma glucose 7.8–11.1 mmol/L measured 120 minutes after ingestion of 75 g glucose in the oral glucose tolerance test]).

Among the women with PCOS, 19,425 were defined based on the PCOS Read code, while the remainder (*n* = 43,785) were defined based on a PCO Read code. In total, 20,162 women had a diagnosis after joining the general practice and were categorized as incident cases. Among women with PCOS, 34.5% of women had documented clinical features suggestive of androgen excess (acne 21.7%, hirsutism 12.9%, and androgenetic alopecia 6.2%), and 29.3% had features suggestive of oligoanovulation (Table 2). Before study entry or during follow-up, 29.4% of women with PCOS (*n* = 18,583) took an oral contraceptive with an antiandrogenic progestin component (cyproterone acetate 22.6% and drospirenone 12.1%), and 24% (*n* = 15,152) received metformin treatment (Table 2).

**Table 2:** Polycystic ovary syndrome (PCOS) diagnostic features and use of medication.

	All cases and control	
Characteristics	PCOS (*n* = 63,210)	Controls (*n* = 121,064)
	Number (%)	Number (%)
**PCOS features**		
Anovulation	18,514 (29.29)	13,466 (11.12)
Any androgen excess	21,786 (34.47)	25,341 (20.93)
Hirsutism	8,142 (12.88)	1,921 (1.59)
Acne	13,708 (21.69)	19,968 (16.49)
Alopecia	3,895 (6.16)	5,472 (4.52)
Polycystic ovaries	48,369 (76.5)	(0.00)
**Medications**		
OCP including an antiandrogenic progestin component*	18,583 (29.40)	15,564 (12.86)
Cyproterone	14,282 (22.59)	8,767 (7.24)
Drospirenone	7,648 (12.10)	9,302 (7.68)
Other antiandrogen	155 (0.25)	13 (0.01)
Metformin	15,152 (23.97)	2,966 (2.45)
Lipid-modifying drugs	2,722 (4.31)	3,432 (2.83)

OCP, oral contraceptive pill.

*Use of either cocyprindiol or cyproterone acetate or drospirenone. Since some patients were prescribed both cyproterone and drospirenone during the period covered, the number of OCP users is slightly smaller than the arithmetic sum of the numbers of cyproterone and drospirenone users.

### Primary analysis

Women with PCOS were found to have an increased rate of NAFLD in comparison to matched controls without PCOS (HR = 2.38, 95% CI 1.99–2.84, *p* < 0.001). The incidence of NAFLD in women with PCOS was 9.2 per 10,000 person years, while in the matched control group, it was 3.9 per 10,000 person years (Table 3). The estimated hazard ratio remained similar after adjustment for age, Townsend score, BMI, diabetes or IGR, and hypothyroidism at baseline (HR = 2.23, 95% CI 1.86–2.66, *p* < 0.001) and when adjusted for dysglycemia developing during follow-up (HR = 2.14, 95% CI 1.79–2.56, *p* < 0.001) (Table 3).

**Table 3:** Hazard of women with polycystic ovary syndrome (PCOS) developing nonalcoholic fatty liver disease (NAFLD) compared to women without PCOS.

	Primary analysis		Sensitivity analysis (Incident cases)		Sensitivity analysis (PCOS-specific Read codes only)	
	PCOS (Exposed)	Controls (Unexposed)	PCOS (Exposed)	Controls (Unexposed)	PCOS (Exposed)	Controls (Unexposed)
Total number of participants	63,210	121,064	20,162	37,544	19,425	35,967
Person years	297,842	576,065	94,778	167,009	84,041	149,852
Incident NAFLD *n* (%)	274 (0.43)	222 (0.18)	80 (0.40)	65 (0.17)	83 (0.43)	64 (0.18)
Incidence rates per 10,000 person years	9.2	3.9	8.4	3.9	9.9	4.3
Hazard ratio (95% CI)	2.38 (1.99–2.84)		2.15 (1.55–2.99)		2.24 (1.62–3.11)	
*p*-value	<0.001		<0.001		<0.001	
Baseline adjusted hazard ratio (95% CI)*	2.23 (1.86–2.66)		2.07 (1.49–2.87)		2.10 (1.52–2.93)	
*p*-value	<0.001		<0.001		<0.001	
Follow-up adjusted hazard ratio (95% CI)^†^	2.14 (1.79–2.56)		1.98 (1.43–2.75)		2.04 (1.47–2.84)	
*p*-value	<0.001		<0.001		<0.001	

*Adjusted for age, Townsend score, BMI, diabetes or impaired glucose regulation, and hypothyroidism.

^†^Adjusted for age, Townsend score, BMI, diabetes or impaired glucose regulation up to end of follow-up, and hypothyroidism at baseline.

Analysis excluding women with diabetes or IGR at baseline and by censoring when they developed diabetes or IGR did not alter our estimates (HR = 2.22, 95% CI 1.85–2.68, *p* < 0.001) (S5 Table).

Increasing age (HR = 1.05, 95% CI 1.03–1.06, *p* < 0.001), BMI (BMI 25–30 kg/m^2^, HR = 3.37, 95% CI 2.36–4.83, *p* < 0.001; and BMI >30 kg/m^2^, HR = 6.98, 95% CI 5.07–9.60, *p* < 0.001), and diabetes or IGR (HR = 2.39, 95% CI 1.76–3.25, *p* < 0.001) at baseline were also found to be significant independent factors associated with an increased rate of NAFLD (S6 Table), with the impact of PCOS on NAFLD rate estimated to be of a similar magnitude to the impact of diabetes and IGR.

### Sensitivity analysis

Findings were robust when analysis was limited to PCOS codes only (adjusted HR = 2.10, 95% CI 1.52–2.93, *p* < 0.001) and when limited to incident cases only (HR = 2.07, 95% CI 1.49–2.87, *p* < 0.001) (Table 3).

### Analysis to assess if NALFD risk in women with PCOS was independent of BMI

**Fig 2:**
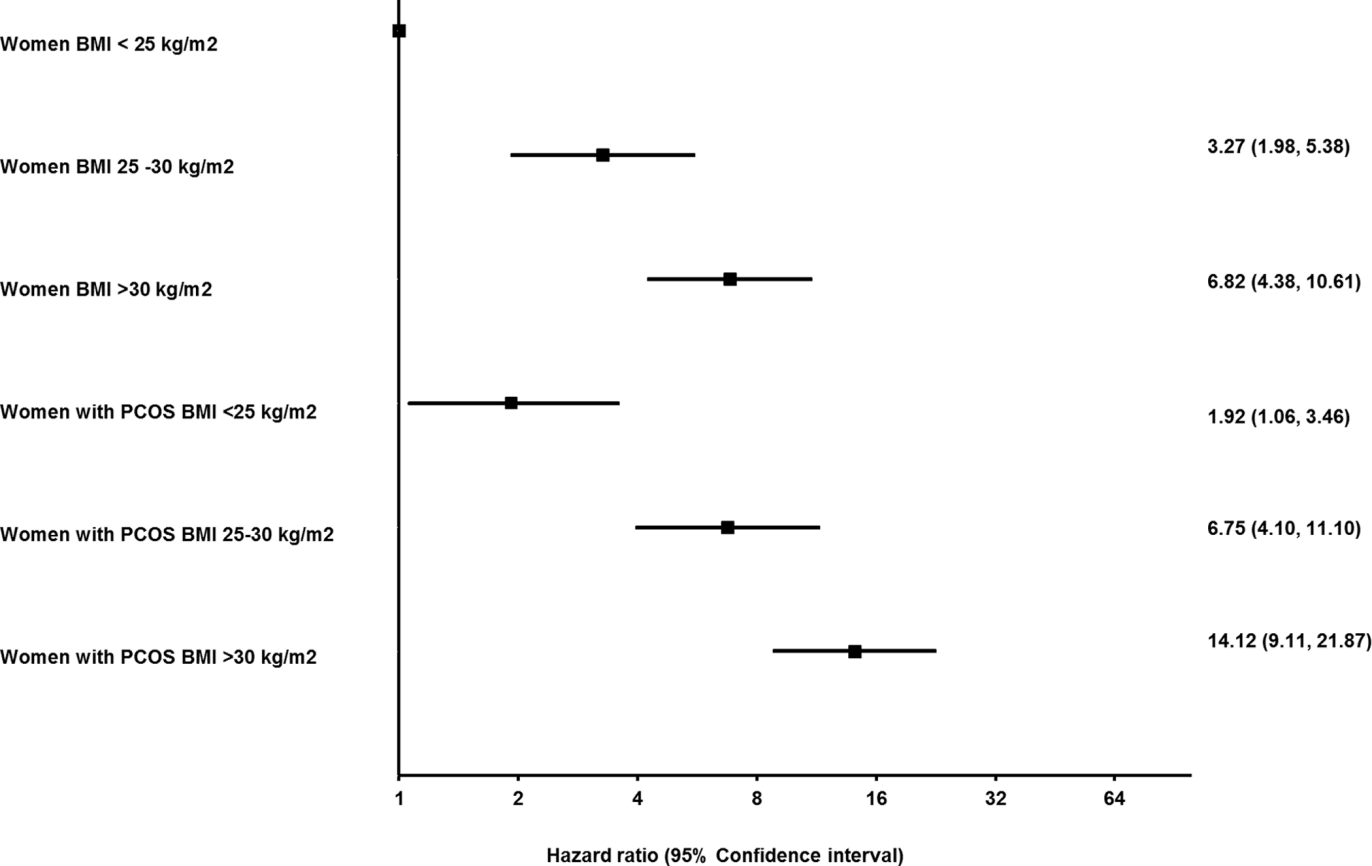
Impact of BMI and polycystic ovary syndrome (PCOS) on the hazard of nonalcoholic fatty liver disease (NAFLD). PCOS and control groups were subcategorized into lean, overweight, and obese groups according to BMI, with lean control women designated as the reference category (unexposed, BMI <25 kg/m^2^). The analysis is adjusted for age, Townsend score of social deprivation, diabetes or impaired glucose regulation, and hypothyroidism at baseline. The hazard of NAFLD increased with BMI in both control women and women with PCOS. Lean women with PCOS had an almost 2-fold higher hazard of NAFLD compared to lean controls. The highest hazard of developing NAFLD was observed in the obese PCOS cohort.

In the subgroup analysis, women with PCOS were at increased risk of NAFLD in all 3 BMI subgroups (HR = 1.85, 95% CI 1.02–3.34, *p* = 0.043 in <25 kg/m^2^; HR = 2.05, 95% CI 1.37–3.07, *p* < 0.001 for 25–30 kg/m^2^; and HR = 2.06, 95% CI 1.65–2.57, *p* < 0.001 for >30 kg/m^2^) (S7 Table). In the analysis with interaction terms for PCOS and BMI, women with PCOS and a BMI of <25 kg/m^2^ had twice the risk of developing NAFLD (HR = 1.92, 95% CI 1.06–3.46, *p* < 0.031), while women with PCOS with a BMI of 25–30 kg/m^2^ or >30 kg/m^2^ were, respectively, 7 times (HR = 6.75, 95% CI 4.10–11.10, *p* < 0.001) and 14 times (HR = 14.12, 95% CI 9.11–21.87, *p* < 0.001) more at risk compared to control women with a BMI less than 25 kg/m^2^ (Fig 2 and S8 Table).

### Analysis to explore if androgen excess in women with PCOS confers a riagaing patients with achalsiag the ting potnetial atients. Future studies should aim to replicate our findings in othersk for NALFD

Introducing androgen excess features into the primary model suggested a potential reduction in the HR from 2.23 (95% CI 1.86–2.66) to 2.07 (95% CI 1.72–2.50) (S9 Table).

#### Clinical features associated with NAFLD in women with PCOS

Along with increasing age, higher BMI and dysglycemia, anovulation (HR = 1.36, 95% CI 1.06–1.756, *p* = 0.02) and clinical androgen excess features, hirsutism (HR = 1.41, 95% CI 1.06–1.870, *p* = 0.01) and alopecia (HR = 1.74, 95% CI 1.23–2.45, *p* = 0.02), but not acne (HR = 0.87, 95% CI 0.62–1.22, *p* = 0.87), were found to be significantly associated with NAFLD within the women with PCOS (Table 4). Antiandrogen therapy was associated with reduced hazard, but this did not reach statistical significance (HR = 0.80, 95% CI 0.59–1.097, *p* = 0.16) (Table 4). Increasing age, Townsend index of deprivation, higher BMI, hypothyroidism, and anovulation (HR = 1.60, 95% CI 1.16–2.20, *p* = 0.004) were found to be significantly associated with NAFD within women without PCOS (S10 Table).

**Table 4:** Factors associated with nonalcoholic fatty liver disease (NAFLD) amongst women with polycystic ovary syndrome (PCOS) (*n* = 63,210).

Covariates	Hazard ratio	95% CI	*p*-value
**Age (years)**	1.04	(1.02–1.06)	<0.001
**Townsend**			
1	1.00		
2	0.97	(0.66–1.43)	0.88
3	0.86	(0.59–1.28)	0.46
4	1.25	(0.87–1.79)	0.23
5	1.16	(0.78–1.74)	0.47
Missing	1.18	(0.68–2.07)	0.56
**BMI (kg/m**^**2**^**) category**			
<25	1.00		
25–30	3.46	(2.07–5.78)	<0.001
>30	6.91	(4.35–10.98)	<0.001
Missing	3.05	(1.76–5.30)	<0.001
Diabetes or IGR*	2.57	(1.65–4.01)	<0.001
**Hypothyroidism**	1.04	(0.63–1.74)	0.87
**Anovulation**	1.36	(1.06–1.75)	0.02
**Androgen excess feature**			
**Hirsutism**	1.41	(1.06–1.87)	0.01
**Acne**	0.87	(0.62–1.22)	0.87
**Alopecia**	1.74	(1.23–2.45)	0.02
**Polycystic ovaries**	1.08	(0.79–1.49)	0.61
**Lipid-modifying drugs**	1.26	(0.71–2.23)	0.43
**Metformin**	1.01	(0.74–1.39)	0.94
**Antiandrogen drug**	0.80	(0.59–1.09)	0.16

*IGR, impaired glucose regulation (includes impaired fasting glucose [IFG; fasting plasma glucose 6.1–6.9 mmol/L] and impaired glucose tolerance [IGT; plasma glucose 7.8–11.1 mmol/L measured 120 minutes after ingestion of 75 g glucose in the oral glucose tolerance test]).

### Analysis of the cohorts with serum testosterone and SHBG measurements

**Fig 3:**
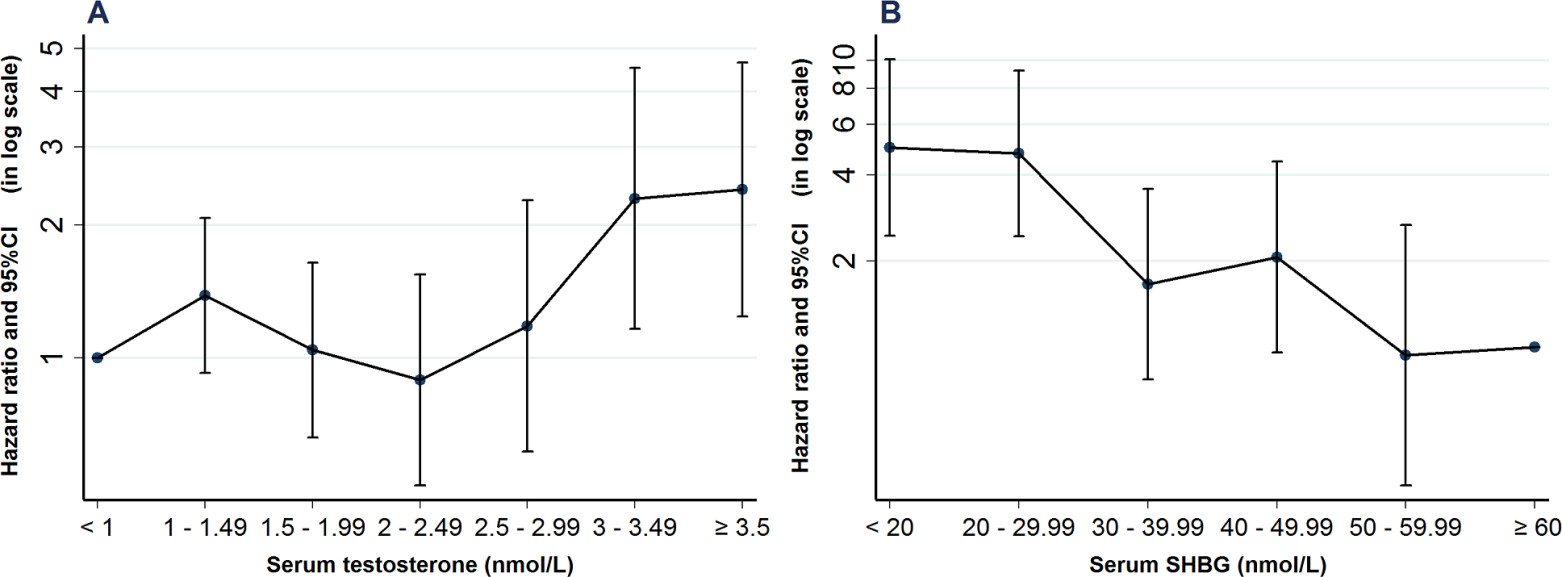
Hazard of nonalcoholic fatty liver disease (NAFLD) according to serum testosterone and sex hormone-binding globulin (SHBG) levels. (A) Serum testosterone (nmol/L) was available in 71,061 unselected women. Using women with testosterone levels below 1.0 nmol/L as a reference cohort, the hazard of NAFLD was 2.4-fold higher in those with serum testosterone levels of >3 nmol/l. (B) Serum SHBG was available in 49,625 unselected women. Using a reference cohort with SHBG levels of ≥60 nmol/L, the hazard of NAFLD was almost 5-fold elevated in patients with serum levels of <30 nmol/L. Both analyses are adjusted for age, Townsend score, diabetes or impaired glucose regulation, and hypothyroidism at baseline.

There were 71,061 eligible women with a measurement of serum testosterone (median age 31.8 years, IQR 25.9–38.1 years) (S2 Fig). Within this cohort, serum testosterone results (nmol/l) were distributed as follows (concentration, %): <1, 33.7%; 1–1.49, 24.9%; 1.5–1.99, 19.0%; 2–2.49, 11.5%; 2.5–2.99, 5.7%; 3–3.49, 2.6%; and ≥3.5, 2.6% (S11 Table). Patients in both the 3–3.49 nmol/l (adjusted HR = 2.30, 95% CI 1.16–4.53, *p* = 0.017) and ≥3.5 nmol/l (adjusted HR = 2.40, 95% CI 1.24–4.66, *p* = 0.009) groups had increased rates of NAFLD (Fig 3A and S12–S14 Tables).

There were 49,625 eligible women with a measurement of serum SHBG (median age 30.4 years, IQR 24.9–36.6 years) (S2 Fig). Within this cohort, there were 9.2% in the <20 nmol/L group, 15.3% in the 20–29.99 group, 16.0% in the 30–39.99, 14.4% in the 40–49.99, 11.7% in the 50–59.99 and 33.6% in the ≥60 nmol/L groups (S15 Table). Patients with lower SHBG (<30 nmol/L: adjusted HR = 4.98, 95% CI 2.45–10.11, *p* < 0.001; <20 nmol/L; adjusted HR = 4.75, 95% CI 2.44–9.25, *p* < 0.001) had increased rates of NAFLD (Fig 3B, S16 and S17 Tables).

## Discussion

The results of this study provide, to our knowledge, the most conclusive evidence to date that women with PCOS have an increased rate of NAFLD above that conferred by simple obesity. A previous meta-analysis of 7 studies with a combined sample size of less than 600 patients suggested an almost 4-fold increase in the risk of NAFLD in women with PCOS compared with matched controls [13]. A very recent meta-analysis that combined data of 2,700 patients from 17 studies found a 2.5-fold increased risk of NAFLD in women with PCOS compared with age- and BMI-matched controls [19]. To our knowledge, our population-based longitudinal cohort study is the first longitudinal and by far the largest study to examine the association between PCOS and NAFLD, analyzing over 63,000 women with PCOS against 121,000 matched controls. A diagnosis of PCOS was consistently associated with a 2.0–2.4-fold increase in rate of NAFLD in multiple adjusted analyses, and this increased hazard persisted even when restricting to patients with PCOS. Intriguingly, even women with PCOS with a normal BMI had a significantly increased rate of NAFLD.

Within our PCOS cohort, androgen excess, anovulation, and dysglycemia independently increased the hazard of developing NAFLD. The prevalence of androgen excess in our study is likely to be an underestimate, as multiple studies have shown prevalence estimates as high as 80% in PCOS [5]. To mitigate this, we further probed the association between androgens and NAFLD hazard by examining the impact of serum testosterone and SHBG levels on rate of NAFLD in separate independent cohorts. The analysis of these 2 cohorts demonstrated that female testosterone levels in excess of 3 nmol/L and SHBG levels below 30 nmol/L were associated with an increased rate of NAFLD.

The mechanisms underpinning the increased NAFLD rate in PCOS are likely to represent a complex interplay between androgen excess, insulin resistance, and obesity [37]. However, our data provide convincing evidence for an independent contribution from androgen excess. Several smaller studies have previously implicated androgen excess as associated with metabolic dysfunction in women with PCOS [16,38,39]. Increasing free serum testosterone levels in premenopausal women was associated with NAFLD in midlife in a recent prospective study, independent of traditional risk factors such as insulin resistance, BMI, and dyslipidemia [40]. Similarly, a meta-analysis looking at 2,700 patients from 17 studies found that serum testosterone was higher in those women with PCOS who had NAFLD [19].

We have recently provided mechanistic evidence to the association between PCOS, androgen excess, and NAFLD by showing that intra-adipose androgen generation in PCOS drives systemic lipotoxicity by increasing adipocyte hypertrophy and fatty acid overspill [41]. We found that PCOS patients with androgen excess had increased circulating glycerophospholipids and lysoglycerophospholipids, recently identified as potential biomarkers of NASH [42]. We also observed that systemic lipotoxicity increased further after an acute androgen challenge in patients with PCOS, but not in BMI-matched controls [41]. Androgen-mediated adipose lipotoxicity could therefore represent an important mechanism conveying liver injury in hyperandrogenemic PCOS.

Previous in vitro data have suggested a direct effect of androgens on hepatic lipid metabolism in women, with testosterone increasing lipogenic gene expression and de novo lipogenesis in primary human hepatocytes from female, but not male, donors [43]. The sexually dimorphic role of androgens in metabolic disease is an emerging topic, with female androgen excess and male hypogonadism sharing an overlapping metabolic phenotype characterized by abdominal obesity, dyslipidemia, insulin resistance, and NAFLD [44]. Mirroring our data, a recent meta-analysis of 13,721 men and 5,840 women demonstrated a significant sexually dimorphic association between serum testosterone and NAFLD risk [45]. Higher testosterone levels reduced the likelihood of NAFLD in men but conversely increased the risk in women. The same meta-analysis concluded that increasing SHBG concentrations in both sexes independently lowered the risk of hepatic steatosis. In our study, serum SHBG levels below 30 nmol/L, as typically found in androgen excess, conferred an almost 5-fold increase in the hazard of NAFLD. The majority of circulating testosterone is bound to SHBG in men and women, and it is generally accepted that only unbound or “free” testosterone is able to enter metabolic target tissues [46]. Hepatic SHBG output is suppressed by insulin [5], and therefore, decreased circulating SHBG is a surrogate marker of hyperinsulinemia in the setting of systemic insulin resistance [47]. SHBG metabolism in women therefore sits at the interface between insulin resistance and androgen excess, both key players in PCOS-related metabolic dysfunction [48]. However, it should be noted that interpreting the impact of decreased serum SHBG concentrations on NAFLD risk in women is limited by the fact that both factors known to be associated with decreased SHBG, androgen excess and insulin resistance, are highly prevalent in women with PCOS [49,50].

Insulin resistance is implicated as a major player in the pathogenesis of NAFLD, and impaired suppression of adipose tissue lipolysis by insulin may lead directly to systemic free fatty acid overspill from adipocytes, with accumulation of intrahepatic diacylglycerol and triacylglycerol, as well as likely direct hepatocyte injury [51]. Parameters of insulin resistance were not available in our cohort, but we were able to phenotype patients according to glycemic status. The presence of dysglycemia was a significant factor associated with increased hazard of NAFLD within the PCOS population (HR = 2.56), but the elevated rate of NAFLD persisted in our PCOS cohort compared to control women even after correction for glycemic status at baseline and during follow-up.

The effect of obesity on NAFLD rate in this study also merits discussion. Multivariate analysis consistently showed that a diagnosis of PCOS conferred an increased rate of NAFLD independent of body weight. The observation of a significantly increased adjusted HR of NAFLD in lean PCOS patients is notable, as at present these patients are not perceived to be at high risk of progression to metabolic dysfunction [52]. As lean women with PCOS exhibit insulin resistance much more rarely than those with obesity [53], it is very reasonable to assume that androgen excess is the major factor driving the increased NAFLD hazard in women of normal weight with PCOS. This lends to the argument that androgen excess is a major causative factor in the development of NAFLD.

The limitations of this large population study include its retrospective nature, limited documentation of the diagnostic criteria, clinical features and investigations used for identifying PCOS and NAFLD, absence of any information on the stage of NAFLD, and the fact that no data were available on laboratory assays employed to measure serum testosterone concentrations. It is likely that a proportion of serum testosterone values were not measured by gold-standard liquid chromatography-tandem mass spectrometry techniques. Furthermore, data on the serum androgen precursor androstenedione, previously highlighted as the most sensitive biomarker of androgen burden and PCOS-related metabolic dysfunction [5], were not available. In addition, patients who underwent serum testosterone and SHBG measurements are not representative of the general population, as they were likely investigated for clinical indications such as suspected PCOS. It is possible that women in our 2 independent cohorts with biochemical results for serum testosterone and SHBG indicative of androgen excess were indeed individuals with underlying PCOS who may not have been evaluated further and given a diagnosis of PCOS. As expected in a study of this nature, there were no indices of insulin resistance available in the patient cohorts.

In conclusion, we found an increased rate of NAFLD in PCOS, and this extended to lean women with PCOS, indicative of androgen excess as a likely major causal factor. We could show that increased circulating androgen burden determined by serum testosterone and reduced SHBG levels independently confer an increased rate of NAFLD. Of note, antiandrogen treatment was associated with a reduction in NAFLD rate, though this failed to reach statistical significance, likely due to the limited number of women on antiandrogens in our cohort.

Our data support that androgen excess is an independent adverse metabolic risk factor in PCOS and that metabolic risk stratification in PCOS should take androgen excess into account. We suggest that women with PCOS should be considered for systematic screening for NAFLD, if biochemical evidence of androgen excess is present; the diagnostic work-up should be carried out in accordance with clinical guidelines for the assessment of individuals deemed at increased risk of NAFLD [54]. Future studies need to clarify whether reducing androgen burden will convey long-term metabolic benefits in both lean and obese women with PCOS with androgen excess, including a reduction in their risk of developing NAFLD.

## Supporting information

S1 Fig Visual representation of study timeline and selection process (identification of the exposed group [individuals with polycystic ovary syndrome (PCOS)/polycystic ovaries (PCO) read codes] and their controls [without PCOS/PCO] matched 1:2 by age at index date, BMI, and practice of origin). (PNG)

S2 Fig Venn diagram showing the overlap between polycystic ovary syndrome (PCOS)/polycystic ovaries (PCO), testosterone, and sex hormone-binding globulin (SHBG) cohorts. (JPG)

S1 Table NAFLD among women with polycystic ovary syndrome (PCOS)/polycystic ovaries (PCO) by serum testosterone concentration category (*n* = 11,251). (DOCX)

S2 Table NAFLD among women with polycystic ovary syndrome (PCOS)/polycystic ovaries (PCO) by serum sex hormone-binding globulin (SHBG) concentration category (*n* = 10,827). (DOCX)

S3 Table Read codes used in data extraction. (DOCX)

S4 Table Drug codes used to identify antiandrogens, lipid-modifying drugs, and metformin. (DOCX)

S5 Table Hazard of women with polycystic ovary syndrome (PCOS) developing nonalcoholic fatty liver disease (NAFLD) compared to women without PCOS using diabetes censored data. (DOCX)

S6 Table Regression model estimates for hazard of women with polycystic ovary syndrome (PCOS) developing nonalcoholic fatty liver disease (NAFLD) compared to women without PCOS (*n* = 184,274). (DOCX)

S7 Table Subgroup analysis for hazard of women with polycystic ovary syndrome (PCOS) developing nonalcoholic fatty liver disease (NAFLD) compared to women without PCOS stratified by BMI category. (DOCX)

S8 Table Regression model estimates for impact of BMI and polycystic ovary syndrome (PCOS) on hazard of women with PCOS developing nonalcoholic fatty liver disease (NAFLD) compared to women without PCOS (*n* = 184,274). (DOCX)

S9 Table Regression model estimates for hazard of women with polycystic ovary syndrome (PCOS) developing nonalcoholic fatty liver disease (NAFLD) compared to women without PCOS with addition of PCOS diagnostic features as predictor (*n* = 184,274). (DOCX)

S10 Table Factors associated with nonalcoholic fatty liver disease (NAFLD) amongst women without polycystic ovary syndrome (PCOS) (*n* = 121.064). (DOCX)

S11 Table Baseline characteristics in cohort of women with available serum testosterone measurement (*n* = 71,061). (DOCX)

S12 Table Factors associated with nonalcoholic fatty liver disease (NAFLD) amongst the cohort of women with available serum testosterone measurement (*n* = 71,061). (DOCX)

S13 Table Hazard of women with a serum testosterone level of ≥1 nmol/L developing nonalcoholic fatty liver disease (NAFLD) compared to women with a serum testosterone level of <1 nmol/L (*n* = 71,061). (DOCX)

S14 Table Risk of nonalcoholic fatty liver disease (NAFLD) in women with available serum testosterone measurement following exclusion of participants with PCOS/PCO codes (*n* = 58,606). (DOCX)

S15 Table Baseline characteristics in the cohort of women with available serum sex hormone-binding globulin (SHBG) measurement (n = 49,625). (DOCX)

S16 Table Factors associated with nonalcoholic fatty liver disease (NAFLD) amongst the cohort of women with available serum sex hormone-binding globulin (SHBG) measurement (*n* = 49,625). (DOCX)

S17 Table Hazard of women with a serum sex hormone-binding globulin (SHBG) level of <60 nmol/L developing NAFLD compared to women with a serum sex hormone-binding globulin (SHBG) level of ≥60 nmol/L (*n* = 49,625). (DOCX)

S18 Table Risk of nonalcoholic fatty liver disease (NAFLD) based on available serum sex hormone-binding globulin (SHBG) measurement following exclusion of participants with polycystic ovary syndrome (PCOS)/polycystic ovaries (PCO) codes (*n* = 38,784). (DOCX)

S1 Text Analysis plan extracted from the approved protocol. (DOCX)

S2 Text The reporting of studies conducted using observational routinely collected health data (RECORD) statement—checklist of items, extended from the strengthening the reporting of observational studies in epidemiology (STROBE) statement, that should be reported in observational studies using routinely collected health data. (DOCX)

## Abbreviations

BMI: body mass index
HR: hazard ratio
IFG: impaired fasting glucose
IGR: impaired glucose regulation
IGT: impaired glucose tolerance
NAFLD: nonalcoholic fatty liver disease
NASH: nonalcoholic steatohepatitis
PCO: polycystic ovaries
PCOS: polycystic ovary syndrome
SHBG: sex hormone-binding globulin
RECORD: Reporting of Studies Conducted Using Observational Routinely Collected Health Data
STROBE: Strengthening the Reporting of Observational Studies in Epidemiology
