# Supplementary figures and images for: Polycystic ovary syndrome, androgen excess, and the risk of nonalcoholic fatty liver disease in women: A longitudinal study based on a United Kingdom primary care database
Source: PLoS Med. 2018 Mar 28;15(3):e1002542. doi: 10.1371/journal.pmed.1002542 (PMC5873722; doi:10.1371/journal.pmed.1002542)

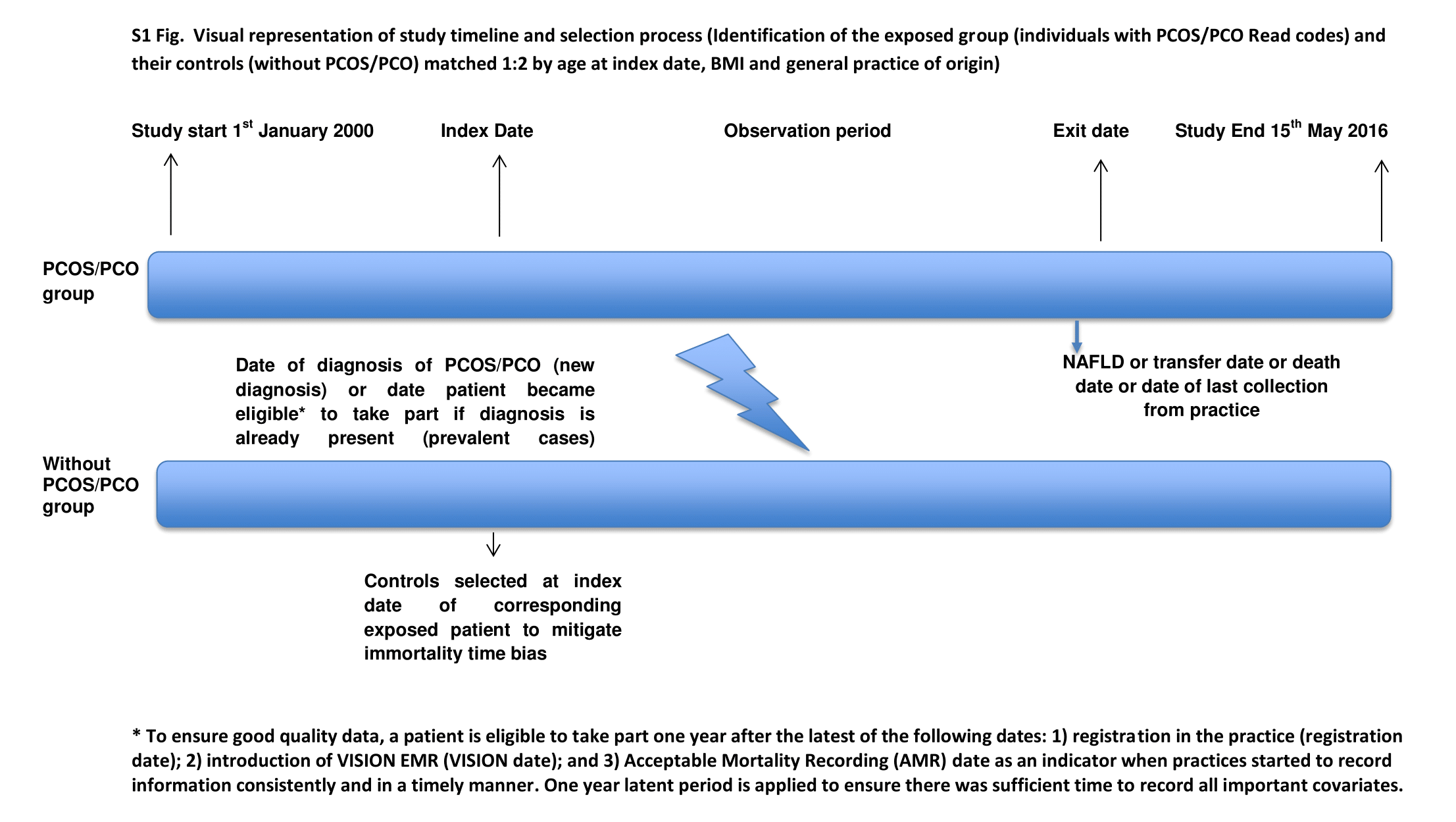

Supplement: S1 Fig — (PNG) [file pmed.1002542.s001.png]

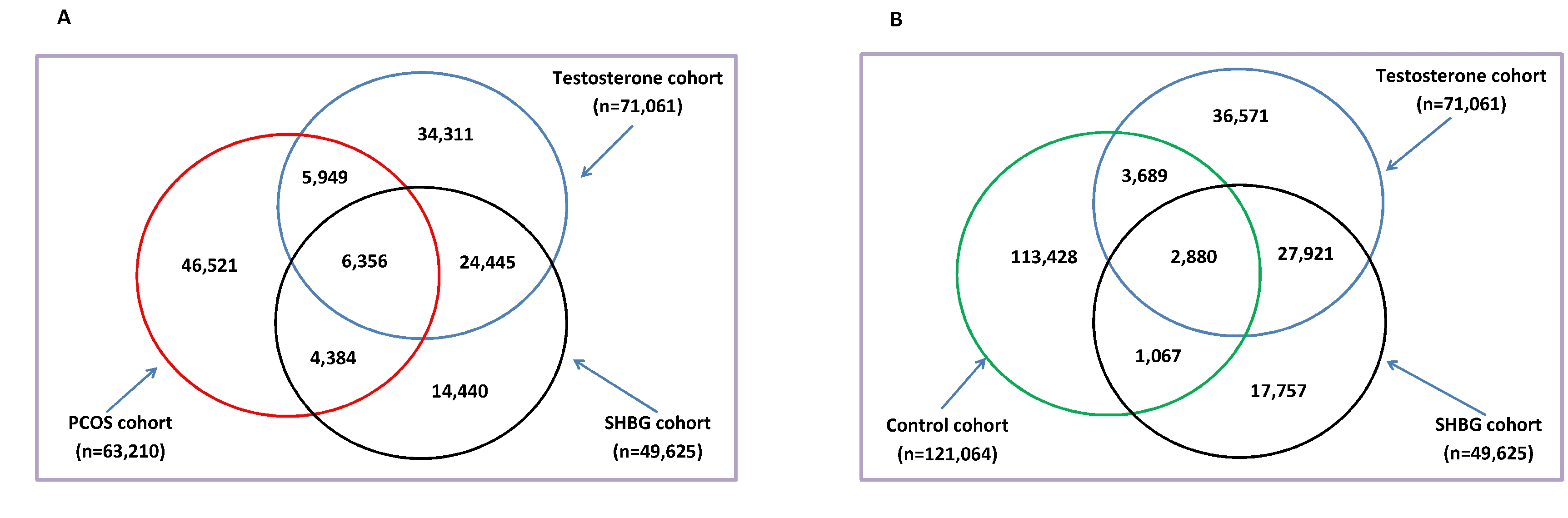

Supplement: S2 Fig — (JPG) [file pmed.1002542.s002.jpg]
