# Supplementary material for: Polycystic ovary syndrome, androgen excess, and the risk of nonalcoholic fatty liver disease in women: A longitudinal study based on a United Kingdom primary care database
Source: PLoS Med. 2018 Mar 28;15(3):e1002542. doi: 10.1371/journal.pmed.1002542 (PMC5873722; doi:10.1371/journal.pmed.1002542)
Supplement: S1 Table — (DOCX) [file pmed.1002542.s003.docx]

**S1: NAFLD among PCOS/PCO women by serum testosterone concentration category (n=11,251)**

|  | **Serum testosterone concentration categories (nmol/L)** | | | | | | |
| --- | --- | --- | --- | --- | --- | --- | --- |
|  | **< 1** | **1 - 1.49** | **1.5 - 1.99** | **2 - 2.49** | **2.5 - 2.99** | **3 - 3.49** | **≥ 3.5** |
| Number of participants | 1,873 | 2,174 | 2,372 | 1,941 | 1,266 | 740 | 824 |
| Incident NAFLD | 9 | 13 | 15 | 5 | 6 | 7 | 6 |
| Person years | 10,472 | 10,805 | 12,093 | 10,397 | 6,684 | 4,048 | 4,650 |
| Incidence rate (per 10,000 person years) | 8.59 | 12.03 | 12.4 | 4.81 | 8.98 | 17.29 | 12.9 |
| Hazard Ratio | 1.0 | 1.42 | 1.47 | 0.57 | 1.07 | 2.08 | 1.52 |
| 95% CI of Hazard ratio |  | (0.61, 3.33) | (0.64, 3.37) | (.19, 1.70) | (0.38, 2.99) | (0.77, 5.59) | (0.54, 4.28) |
| p-value |  | 0.415 | 0.359 | 0.311 | 0.905 | 0.147 | 0.426 |
