# Supplementary material for: Polycystic ovary syndrome, androgen excess, and the risk of nonalcoholic fatty liver disease in women: A longitudinal study based on a United Kingdom primary care database
Source: PLoS Med. 2018 Mar 28;15(3):e1002542. doi: 10.1371/journal.pmed.1002542 (PMC5873722; doi:10.1371/journal.pmed.1002542)
Supplement: S2 Table — (DOCX) [file pmed.1002542.s004.docx]

S2: NAFLD among PCOS/PCO women by serum SHBG concentration category (n=10,827)

|  | **Serum SHBG concentration categories (nmol/L)** | | | | | |
| --- | --- | --- | --- | --- | --- | --- |
|  | **<20** | **20 - 29.99** | **30 - 39.99** | **40 - 49.99** | **50 - 59.99** | **≥60** |
| Number of participants | 1,955 | 2,497 | 1,855 | 1,364 | 924 | 2,232 |
| Incident NAFLD | 13 | 15 | 4 | 5 | 1 | 3 |
| Person years | 7,675 | 9,797 | 7,261 | 5,269 | 3,441 | 7,753 |
| Incidence rate (per 10,000 person years) | 16.94 | 15.31 | 5.51 | 9.49 | 2.91 | 3.87 |
| Hazard Ratio | 4.43 | 3.99 | 1.44 | 2.45 | 0.75 | 1.0 |
| 95% CI of Hazard ratio | (1.26, 15.53) | (1.15, 13.78) | (0.32, 6.43) | (0.59, 10.27) | (0.08, 7.23) | - |
| p-value | 0.02 | 0.03 | 0.63 | 0.22 | 0.80 | - |
