# Supplementary material for: Polycystic ovary syndrome, androgen excess, and the risk of nonalcoholic fatty liver disease in women: A longitudinal study based on a United Kingdom primary care database
Source: PLoS Med. 2018 Mar 28;15(3):e1002542. doi: 10.1371/journal.pmed.1002542 (PMC5873722; doi:10.1371/journal.pmed.1002542)
Supplement: S3 Table — (DOCX) [file pmed.1002542.s005.docx]

S3: Read codes used in data extraction

| **Condition** | **Read Code** | **Description** |
| --- | --- | --- |
| Polycystic ovary syndrome | C164.12 | Stein - Leventhal syndrome |
|  | C165.00 | Polycystic ovarian syndrome |
|  | 7E25300 | Endoscopic drilling of ovary |
| Polycystic ovaries | C164.00 | Polycystic ovaries |
|  | C164.13 | Multi-cystic ovaries |
| Non- alcoholic fatty liver disease | J61y800 | Non-alcoholic steatohepatitis |
|  | J61y100 | Non-alcoholic fatty liver |
| Ovulatory dysfunction | 1571 | H/O: amenorrhoea |
|  | K590.00 | Absence of menstruation |
|  | K590.11 | Amenorrhoea |
|  | K590100 | Secondary amenorrhoea |
|  | K590z00 | Amenorrhoea NOS |
|  | K591.00 | Scanty or infrequent menstruation |
|  | K591.11 | Infrequent menstruation |
|  | K591000 | Hypomenorrhoea |
|  | K591100 | Oligomenorrhoea |
|  | K591300 | Secondary oligomenorrhoea |
|  | K591z00 | Scanty or infrequent menstruation NOS |
|  | K5B0.00 | Female infertility of anovulatory origin |
|  | K5B0.11 | Anovular cycle |
|  | K5B0100 | Secondary anovulatory infertility |
|  | K5B0z00 | Female infertility of anovulatory origin NOS |
| Alopecia | Myu6300 | [X]Other androgenic alopecia |
|  | M240.00 | Alopecia |
|  | M240z00 | Alopecia NOS |
|  | M240H00 | Alopecia seborrhoeica |
|  | M240000 | Alopecia unspecified |
|  | 1N02.00 | C/O: hair loss |
|  | M240300 | Frontal alopecia of women |
|  | M240012 | Hair loss |
|  | M240200 | Male pattern alopecia |
|  | M240D00 | Marginal alopecia |
|  | 22D7.11 | O/E – alopecia |
|  | 22D4.00 | O/E - loss of hair |
| Hirsutism | 22D8.00 | O/E – hirsutism |
|  | 22D8000 | O/E - facial hair |
|  | M241.00 | Hirsutism - hypertrichosis |

**Table S3 (Continued)**

| **Condition** | **Read codes** | **Description** |
| --- | --- | --- |
| Acne | Myu6F00 | [X]Acne, unspecified |
|  | 2FG5.00 | Acne scar |
|  | 679g000 | Acne management education |
|  | M25y600 | Acne keloid |
|  | M261000 | Acne vulgaris |
|  | M261100 | Acne conglobata |
|  | M261E00 | Acne excoriee des jeunes filles |
|  | M261F00 | Acne fulminans |
|  | M261H00 | Acne keloid |
|  | M261J00 | Acne necrotica |
|  | M261K00 | Acne keloidalis |
|  | M261X00 | Acne, unspecified |
|  | Myu6800 | [X]Other acne |
|  | M261.00 | Other acne |
|  | M261z00 | Other acne NOS |
|  | M261A00 | Pustular acne |

| Diabetes mellitus | C10..00 | Diabetes mellitus |
| --- | --- | --- |
|  | C100.00 | Diabetes mellitus with no mention of complication |
|  | C100000 | Diabetes mellitus, juvenile type, no mention of complication |
|  | C100011 | Insulin dependent diabetes mellitus |
|  | C100100 | Diabetes mellitus, adult onset, no mention of complication |
|  | C100111 | Maturity onset diabetes |
|  | C100112 | Non-insulin dependent diabetes mellitus |
|  | C100z00 | Diabetes mellitus NOS with no mention of complication |
|  | C101.00 | Diabetes mellitus with ketoacidosis |
|  | C101000 | Diabetes mellitus, juvenile type, with ketoacidosis |
|  | C101100 | Diabetes mellitus, adult onset, with ketoacidosis |
|  | C101y00 | Other specified diabetes mellitus with ketoacidosis |
|  | C101z00 | Diabetes mellitus NOS with ketoacidosis |
|  | C102.00 | Diabetes mellitus with hyperosmolar coma |
|  | C102000 | Diabetes mellitus, juvenile type, with hyperosmolar coma |
|  | C102100 | Diabetes mellitus, adult onset, with hyperosmolar coma |
|  | C102z00 | Diabetes mellitus NOS with hyperosmolar coma |
|  | C103.00 | Diabetes mellitus with ketoacidotic coma |
|  | C103000 | Diabetes mellitus, juvenile type, with ketoacidotic coma |
|  | C103100 | Diabetes mellitus, adult onset, with ketoacidotic coma |
|  | C103y00 | Other specified diabetes mellitus with coma |
|  | C103z00 | Diabetes mellitus NOS with ketoacidotic coma |
|  | C104.00 | Diabetes mellitus with renal manifestation |
|  | C104.11 | Diabetic nephropathy |
|  | C104000 | Diabetes mellitus, juvenile type, with renal manifestation |
|  | C104100 | Diabetes mellitus, adult onset, with renal manifestation |

Table S3 (Continued)

| **Condition** | **Read codes** | **Description** |
| --- | --- | --- |
| Diabetes mellitus | C104y00 | Other specified diabetes mellitus with renal complications |
|  | C104z00 | Diabetes mellitus with nephropathy NOS |
|  | C105.00 | Diabetes mellitus with ophthalmic manifestation |
|  | C105000 | Diabetes mellitus, juvenile type, + ophthalmic manifestation |
|  | C105100 | Diabetes mellitus, adult onset, + ophthalmic manifestation |
|  | C105y00 | Other specified diabetes mellitus with ophthalmic complicatn |
|  | C105z00 | Diabetes mellitus NOS with ophthalmic manifestation |
|  | C106.00 | Diabetes mellitus with neurological manifestation |
|  | C106.11 | Diabetic amyotrophy |
|  | C106.12 | Diabetes mellitus with neuropathy |
|  | C106.13 | Diabetes mellitus with polyneuropathy |
|  | C106000 | Diabetes mellitus, juvenile, + neurological manifestation |
|  | C106100 | Diabetes mellitus, adult onset, + neurological manifestation |
|  | C106y00 | Other specified diabetes mellitus with neurological comps |
|  | C106z00 | Diabetes mellitus NOS with neurological manifestation |
|  | C107.00 | Diabetes mellitus with peripheral circulatory disorder |
|  | C107.11 | Diabetes mellitus with gangrene |
|  | C107.12 | Diabetes with gangrene |
|  | C107000 | Diabetes mellitus, juvenile +peripheral circulatory disorder |
|  | C107100 | Diabetes mellitus, adult, + peripheral circulatory disorder |
|  | C107200 | Diabetes mellitus, adult with gangrene |
|  | C107300 | IDDM with peripheral circulatory disorder |
|  | C107400 | NIDDM with peripheral circulatory disorder |
|  | C107y00 | Other specified diabetes mellitus with periph circ comps |
|  | C107z00 | Diabetes mellitus NOS with peripheral circulatory disorder |
|  | C108.00 | Insulin dependent diabetes mellitus |
|  | C108.11 | IDDM-Insulin dependent diabetes mellitus |
|  | C108.12 | Type 1 diabetes mellitus |
|  | C108.13 | Type I diabetes mellitus |
|  | C108000 | Insulin-dependent diabetes mellitus with renal complications |
|  | C108011 | Type I diabetes mellitus with renal complications |
|  | C108012 | Type 1 diabetes mellitus with renal complications |
|  | C108100 | Insulin-dependent diabetes mellitus with ophthalmic comps |
|  | C108111 | Type I diabetes mellitus with ophthalmic complications |
|  | C108112 | Type 1 diabetes mellitus with ophthalmic complications |
|  | C108200 | Insulin-dependent diabetes mellitus with neurological comps |
|  | C108211 | Type I diabetes mellitus with neurological complications |
|  | C108212 | Type 1 diabetes mellitus with neurological complications |
|  | C108300 | Insulin dependent diabetes mellitus with multiple complicatn |
|  | C108311 | Type I diabetes mellitus with multiple complications |
|  | C108312 | Type 1 diabetes mellitus with multiple complications |
|  | C108400 | Unstable insulin dependent diabetes mellitus |

Table S3 (Continued)

| **Condition** | **Read codes** | **Description** |
| --- | --- | --- |
| Diabetes mellitus | C108411 | Unstable type I diabetes mellitus |
|  | C108412 | Unstable type 1 diabetes mellitus |
|  | C108500 | Insulin dependent diabetes mellitus with ulcer |
|  | C108511 | Type I diabetes mellitus with ulcer |
|  | C108512 | Type 1 diabetes mellitus with ulcer |
|  | C108600 | Insulin dependent diabetes mellitus with gangrene |
|  | C108611 | Type I diabetes mellitus with gangrene |
|  | C108612 | Type 1 diabetes mellitus with gangrene |
|  | C108700 | Insulin dependent diabetes mellitus with retinopathy |
|  | C108711 | Type I diabetes mellitus with retinopathy |
|  | C108712 | Type 1 diabetes mellitus with retinopathy |
|  | C108800 | Insulin dependent diabetes mellitus - poor control |
|  | C108811 | Type I diabetes mellitus - poor control |
|  | C108812 | Type 1 diabetes mellitus - poor control |
|  | C108900 | Insulin dependent diabetes maturity onset |
|  | C108911 | Type I diabetes mellitus maturity onset |
|  | C108912 | Type 1 diabetes mellitus maturity onset |
|  | C108A00 | Insulin-dependent diabetes without complication |
|  | C108A11 | Type I diabetes mellitus without complication |
|  | C108A12 | Type 1 diabetes mellitus without complication |
|  | C108B00 | Insulin dependent diabetes mellitus with mononeuropathy |
|  | C108B11 | Type I diabetes mellitus with mononeuropathy |
|  | C108B12 | Type 1 diabetes mellitus with mononeuropathy |
|  | C108C00 | Insulin dependent diabetes mellitus with polyneuropathy |
|  | C108C11 | Type I diabetes mellitus with polyneuropathy |
|  | C108C12 | Type 1 diabetes mellitus with polyneuropathy |
|  | C108D00 | Insulin dependent diabetes mellitus with nephropathy |
|  | C108D11 | Type I diabetes mellitus with nephropathy |
|  | C108D12 | Type 1 diabetes mellitus with nephropathy |
|  | C108E00 | Insulin dependent diabetes mellitus with hypoglycaemic coma |
|  | C108E11 | Type I diabetes mellitus with hypoglycaemic coma |
|  | C108E12 | Type 1 diabetes mellitus with hypoglycaemic coma |
|  | C108F00 | Insulin dependent diabetes mellitus with diabetic cataract |
|  | C108F11 | Type I diabetes mellitus with diabetic cataract |
|  | C108F12 | Type 1 diabetes mellitus with diabetic cataract |
|  | C108G00 | Insulin dependent diab mell with peripheral angiopathy |
|  | C108G11 | Type I diabetes mellitus with peripheral angiopathy |
|  | C108G12 | Type 1 diabetes mellitus with peripheral angiopathy |
|  | C108H00 | Insulin dependent diabetes mellitus with arthropathy |
|  | C108H11 | Type I diabetes mellitus with arthropathy |
|  | C108H12 | Type 1 diabetes mellitus with arthropathy |
|  | C108J00 | Insulin dependent diab mell with neuropathic arthropathy |

Table S3 (Continued)

| **Condition** | **Read codes** | **Description** |
| --- | --- | --- |
| Diabetes mellitus | C108J11 | Type I diabetes mellitus with neuropathic arthropathy |
|  | C108J12 | Type 1 diabetes mellitus with neuropathic arthropathy |
|  | C108y00 | Other specified diabetes mellitus with multiple comps |
|  | C108z00 | Unspecified diabetes mellitus with multiple complications |
|  | C109.00 | Non-insulin dependent diabetes mellitus |
|  | C109.11 | NIDDM - Non-insulin dependent diabetes mellitus |
|  | C109.12 | Type 2 diabetes mellitus |
|  | C109.13 | Type II diabetes mellitus |
|  | C109000 | Non-insulin-dependent diabetes mellitus with renal comps |
|  | C109011 | Type II diabetes mellitus with renal complications |
|  | C109012 | Type 2 diabetes mellitus with renal complications |
|  | C109100 | Non-insulin-dependent diabetes mellitus with ophthalm comps |
|  | C109111 | Type II diabetes mellitus with ophthalmic complications |
|  | C109112 | Type 2 diabetes mellitus with ophthalmic complications |
|  | C109200 | Non-insulin-dependent diabetes mellitus with neuro comps |
|  | C109211 | Type II diabetes mellitus with neurological complications |
|  | C109212 | Type 2 diabetes mellitus with neurological complications |
|  | C109300 | Non-insulin-dependent diabetes mellitus with multiple comps |
|  | C109311 | Type II diabetes mellitus with multiple complications |
|  | C109312 | Type 2 diabetes mellitus with multiple complications |
|  | C109400 | Non-insulin dependent diabetes mellitus with ulcer |
|  | C109411 | Type II diabetes mellitus with ulcer |
|  | C109412 | Type 2 diabetes mellitus with ulcer |
|  | C109500 | Non-insulin dependent diabetes mellitus with gangrene |
|  | C109511 | Type II diabetes mellitus with gangrene |
|  | C109512 | Type 2 diabetes mellitus with gangrene |
|  | C109600 | Non-insulin-dependent diabetes mellitus with retinopathy |
|  | C109611 | Type II diabetes mellitus with retinopathy |
|  | C109612 | Type 2 diabetes mellitus with retinopathy |
|  | C109700 | Non-insulin dependent diabetes mellitus - poor control |
|  | C109711 | Type II diabetes mellitus - poor control |
|  | C109712 | Type 2 diabetes mellitus - poor control |
|  | C109800 | Reaven's syndrome |
|  | C109900 | Non-insulin-dependent diabetes mellitus without complication |
|  | C109911 | Type II diabetes mellitus without complication |
|  | C109912 | Type 2 diabetes mellitus without complication |
|  | C109A00 | Non-insulin dependent diabetes mellitus with mononeuropathy |
|  | C109A11 | Type II diabetes mellitus with mononeuropathy |
|  | C109A12 | Type 2 diabetes mellitus with mononeuropathy |
|  | C109B00 | Non-insulin dependent diabetes mellitus with polyneuropathy |
|  | C109B11 | Type II diabetes mellitus with polyneuropathy |
|  | C109B12 | Type 2 diabetes mellitus with polyneuropathy |

Table S3 (Continued)

| **Condition** | **Read codes** | **Description** |
| --- | --- | --- |
| Diabetes mellitus | C109C00 | Non-insulin dependent diabetes mellitus with nephropathy |
|  | C109C11 | Type II diabetes mellitus with nephropathy |
|  | C109C12 | Type 2 diabetes mellitus with nephropathy |
|  | C109D00 | Non-insulin dependent diabetes mellitus with hypoglyca coma |
|  | C109D11 | Type II diabetes mellitus with hypoglycaemic coma |
|  | C109D12 | Type 2 diabetes mellitus with hypoglycaemic coma |
|  | C109E00 | Non-insulin depend diabetes mellitus with diabetic cataract |
|  | C109E11 | Type II diabetes mellitus with diabetic cataract |
|  | C109E12 | Type 2 diabetes mellitus with diabetic cataract |
|  | C109F00 | Non-insulin-dependent d m with peripheral angiopath |
|  | C109F11 | Type II diabetes mellitus with peripheral angiopathy |
|  | C109F12 | Type 2 diabetes mellitus with peripheral angiopathy |
|  | C109G00 | Non-insulin dependent diabetes mellitus with arthropathy |
|  | C109G11 | Type II diabetes mellitus with arthropathy |
|  | C109G12 | Type 2 diabetes mellitus with arthropathy |
|  | C109H00 | Non-insulin dependent d m with neuropathic arthropathy |
|  | C109H11 | Type II diabetes mellitus with neuropathic arthropathy |
|  | C109H12 | Type 2 diabetes mellitus with neuropathic arthropathy |
|  | C109J00 | Insulin treated Type 2 diabetes mellitus |
|  | C109J11 | Insulin treated non-insulin dependent diabetes mellitus |
|  | C109J12 | Insulin treated Type II diabetes mellitus |
|  | C109K00 | Hyperosmolar non-ketotic state in type 2 diabetes mellitus |
|  | C10A.00 | Malnutrition-related diabetes mellitus |
|  | C10A.11 | Jamaica type diabetes |
|  | C10A000 | Malnutrition-related diabetes mellitus with coma |
|  | C10A100 | Malnutrition-related diabetes mellitus with ketoacidosis |
|  | C10A200 | Malnutrition-related diabetes mellitus with renal complicatn |
|  | C10A300 | Malnutrit-related diabetes mellitus wth ophthalmic complicat |
|  | C10A400 | Malnutrition-related diabetes mellitus wth neuro complicatns |
|  | C10A500 | Malnutritn-relat diabetes melitus wth periph circul complctn |
|  | C10A600 | Malnutrition-related diabetes mellitus with multiple comps |
|  | C10A700 | Malnutrition-related diabetes mellitus without complications |
|  | C10AW00 | Malnutrit-related diabetes mellitus with unspec complics |
|  | C10AX00 | Malnutrit-relat diabetes mellitus with other spec comps |
|  | C10B.00 | Diabetes mellitus induced by steroids |
|  | C10B000 | Steroid induced diabetes mellitus without complication |
|  | C10C.00 | Diabetes mellitus autosomal dominant |
|  | C10C.11 | Maturity onset diabetes in youth |
|  | C10C.12 | Maturity onset diabetes in youth type 1 |
|  | C10D.00 | Diabetes mellitus autosomal dominant type 2 |
|  | C10D.11 | Maturity onset diabetes in youth type 2 |
|  | C10E.00 | Type 1 diabetes mellitus |

Table S3 (Continued)

| **Condition** | **Read codes** | **Description** |
| --- | --- | --- |
| Diabetes mellitus | C10E.11 | Type I diabetes mellitus |
|  | C10E.12 | Insulin dependent diabetes mellitus |
|  | C10E000 | Type 1 diabetes mellitus with renal complications |
|  | C10E011 | Type I diabetes mellitus with renal complications |
|  | C10E012 | Insulin-dependent diabetes mellitus with renal complications |
|  | C10E100 | Type 1 diabetes mellitus with ophthalmic complications |
|  | C10E111 | Type I diabetes mellitus with ophthalmic complications |
|  | C10E112 | Insulin-dependent diabetes mellitus with ophthalmic comps |
|  | C10E200 | Type 1 diabetes mellitus with neurological complications |
|  | C10E211 | Type I diabetes mellitus with neurological complications |
|  | C10E212 | Insulin-dependent diabetes mellitus with neurological comps |
|  | C10E300 | Type 1 diabetes mellitus with multiple complications |
|  | C10E311 | Type I diabetes mellitus with multiple complications |
|  | C10E312 | Insulin dependent diabetes mellitus with multiple complicat |
|  | C10E400 | Unstable type 1 diabetes mellitus |
|  | C10E411 | Unstable type I diabetes mellitus |
|  | C10E412 | Unstable insulin dependent diabetes mellitus |
|  | C10E500 | Type 1 diabetes mellitus with ulcer |
|  | C10E511 | Type I diabetes mellitus with ulcer |
|  | C10E512 | Insulin dependent diabetes mellitus with ulcer |
|  | C10E600 | Type 1 diabetes mellitus with gangrene |
|  | C10E611 | Type I diabetes mellitus with gangrene |
|  | C10E612 | Insulin dependent diabetes mellitus with gangrene |
|  | C10E700 | Type 1 diabetes mellitus with retinopathy |
|  | C10E711 | Type I diabetes mellitus with retinopathy |
|  | C10E712 | Insulin dependent diabetes mellitus with retinopathy |
|  | C10E800 | Type 1 diabetes mellitus - poor control |
|  | C10E811 | Type I diabetes mellitus - poor control |
|  | C10E812 | Insulin dependent diabetes mellitus - poor control |
|  | C10E900 | Type 1 diabetes mellitus maturity onset |
|  | C10E911 | Type I diabetes mellitus maturity onset |
|  | C10E912 | Insulin dependent diabetes maturity onset |
|  | C10EA00 | Type 1 diabetes mellitus without complication |
|  | C10EA11 | Type I diabetes mellitus without complication |
|  | C10EA12 | Insulin-dependent diabetes without complication |
|  | C10EB00 | Type 1 diabetes mellitus with mononeuropathy |
|  | C10EB11 | Type I diabetes mellitus with mononeuropathy |
|  | C10EB12 | Insulin dependent diabetes mellitus with mononeuropathy |
|  | C10EC00 | Type 1 diabetes mellitus with polyneuropathy |
|  | C10EC11 | Type I diabetes mellitus with polyneuropathy |
|  | C10EC12 | Insulin dependent diabetes mellitus with polyneuropathy |
|  | C10ED00 | Type 1 diabetes mellitus with nephropathy |

Table S3 (Continued)

| **Condition** | **Read codes** | **Description** |
| --- | --- | --- |
| Diabetes mellitus | C10ED11 | Type I diabetes mellitus with nephropathy |
|  | C10ED12 | Insulin dependent diabetes mellitus with nephropathy |
|  | C10EE00 | Type 1 diabetes mellitus with hypoglycaemic coma |
|  | C10EE11 | Type I diabetes mellitus with hypoglycaemic coma |
|  | C10EE12 | Insulin dependent diabetes mellitus with hypoglycaemic coma |
|  | C10EF00 | Type 1 diabetes mellitus with diabetic cataract |
|  | C10EF11 | Type I diabetes mellitus with diabetic cataract |
|  | C10EF12 | Insulin dependent diabetes mellitus with diabetic cataract |
|  | C10EG00 | Type 1 diabetes mellitus with peripheral angiopathy |
|  | C10EG11 | Type I diabetes mellitus with peripheral angiopathy |
|  | C10EG12 | Insulin dependent diab mell with peripheral angiopathy |
|  | C10EH00 | Type 1 diabetes mellitus with arthropathy |
|  | C10EH11 | Type I diabetes mellitus with arthropathy |
|  | C10EH12 | Insulin dependent diabetes mellitus with arthropathy |
|  | C10EJ00 | Type 1 diabetes mellitus with neuropathic arthropathy |
|  | C10EJ11 | Type I diabetes mellitus with neuropathic arthropathy |
|  | C10EJ12 | Insulin dependent diab mell with neuropathic arthropathy |
|  | C10EK00 | Type 1 diabetes mellitus with persistent proteinuria |
|  | C10EK11 | Type I diabetes mellitus with persistent proteinuria |
|  | C10EL00 | Type 1 diabetes mellitus with persistent microalbuminuria |
|  | C10EL11 | Type I diabetes mellitus with persistent microalbuminuria |
|  | C10EM00 | Type 1 diabetes mellitus with ketoacidosis |
|  | C10EM11 | Type I diabetes mellitus with ketoacidosis |
|  | C10EN00 | Type 1 diabetes mellitus with ketoacidotic coma |
|  | C10EN11 | Type I diabetes mellitus with ketoacidotic coma |
|  | C10EP00 | Type 1 diabetes mellitus with exudative maculopathy |
|  | C10EP11 | Type I diabetes mellitus with exudative maculopathy |
|  | C10EQ00 | Type 1 diabetes mellitus with gastroparesis |
|  | C10EQ11 | Type I diabetes mellitus with gastroparesis |
|  | C10ER00 | Latent autoimmune diabetes mellitus in adult |
|  | C10F.00 | Type 2 diabetes mellitus |
|  | C10F.11 | Type II diabetes mellitus |
|  | C10F000 | Type 2 diabetes mellitus with renal complications |
|  | C10F011 | Type II diabetes mellitus with renal complications |
|  | C10F100 | Type 2 diabetes mellitus with ophthalmic complications |
|  | C10F111 | Type II diabetes mellitus with ophthalmic complications |
|  | C10F200 | Type 2 diabetes mellitus with neurological complications |
|  | C10F211 | Type II diabetes mellitus with neurological complications |
|  | C10F300 | Type 2 diabetes mellitus with multiple complications |
|  | C10F311 | Type II diabetes mellitus with multiple complications |
|  | C10F400 | Type 2 diabetes mellitus with ulcer |
|  | C10F411 | Type II diabetes mellitus with ulcer |

Table S3 (Continued)

| **Condition** | **Read codes** | **Description** |
| --- | --- | --- |
| Diabetes mellitus | C10F500 | Type 2 diabetes mellitus with gangrene |
|  | C10F511 | Type II diabetes mellitus with gangrene |
|  | C10F600 | Type 2 diabetes mellitus with retinopathy |
|  | C10F611 | Type II diabetes mellitus with retinopathy |
|  | C10F700 | Type 2 diabetes mellitus - poor control |
|  | C10F711 | Type II diabetes mellitus - poor control |
|  | C10F800 | Reaven's syndrome |
|  | C10F811 | Metabolic syndrome X |
|  | C10F900 | Type 2 diabetes mellitus without complication |
|  | C10F911 | Type II diabetes mellitus without complication |
|  | C10FA00 | Type 2 diabetes mellitus with mononeuropathy |
|  | C10FA11 | Type II diabetes mellitus with mononeuropathy |
|  | C10FB00 | Type 2 diabetes mellitus with polyneuropathy |
|  | C10FB11 | Type II diabetes mellitus with polyneuropathy |
|  | C10FC00 | Type 2 diabetes mellitus with nephropathy |
|  | C10FC11 | Type II diabetes mellitus with nephropathy |
|  | C10FD00 | Type 2 diabetes mellitus with hypoglycaemic coma |
|  | C10FD11 | Type II diabetes mellitus with hypoglycaemic coma |
|  | C10FE00 | Type 2 diabetes mellitus with diabetic cataract |
|  | C10FE11 | Type II diabetes mellitus with diabetic cataract |
|  | C10FF00 | Type 2 diabetes mellitus with peripheral angiopathy |
|  | C10FF11 | Type II diabetes mellitus with peripheral angiopathy |
|  | C10FG00 | Type 2 diabetes mellitus with arthropathy |
|  | C10FG11 | Type II diabetes mellitus with arthropathy |
|  | C10FH00 | Type 2 diabetes mellitus with neuropathic arthropathy |
|  | C10FH11 | Type II diabetes mellitus with neuropathic arthropathy |
|  | C10FJ00 | Insulin treated Type 2 diabetes mellitus |
|  | C10FJ11 | Insulin treated Type II diabetes mellitus |
|  | C10FK00 | Hyperosmolar non-ketotic state in type 2 diabetes mellitus |
|  | C10FK11 | Hyperosmolar non-ketotic state in type II diabetes mellitus |
|  | C10FL00 | Type 2 diabetes mellitus with persistent proteinuria |
|  | C10FL11 | Type II diabetes mellitus with persistent proteinuria |
|  | C10FM00 | Type 2 diabetes mellitus with persistent microalbuminuria |
|  | C10FM11 | Type II diabetes mellitus with persistent microalbuminuria |
|  | C10FN00 | Type 2 diabetes mellitus with ketoacidosis |
|  | C10FN11 | Type II diabetes mellitus with ketoacidosis |
|  | C10FP00 | Type 2 diabetes mellitus with ketoacidotic coma |
|  | C10FP11 | Type II diabetes mellitus with ketoacidotic coma |
|  | C10FQ00 | Type 2 diabetes mellitus with exudative maculopathy |
|  | C10FQ11 | Type II diabetes mellitus with exudative maculopathy |
|  | C10FR00 | Type 2 diabetes mellitus with gastroparesis |
|  | C10FR11 | Type II diabetes mellitus with gastroparesis |

Table S3 (Continued)

| **Condition** | **Read codes** | **Description** |
| --- | --- | --- |
| Diabetes mellitus | C10FS00 | Maternally inherited diabetes mellitus |
|  | C10G.00 | Secondary pancreatic diabetes mellitus |
|  | C10G000 | Secondary pancreatic diabetes mellitus without complication |
|  | C10H.00 | Diabetes mellitus induced by non-steroid drugs |
|  | C10H000 | DM induced by non-steroid drugs without complication |
|  | C10J.00 | Insulin autoimmune syndrome |
|  | C10J000 | Insulin autoimmune syndrome without complication |
|  | C10K.00 | Type A insulin resistance |
|  | C10K000 | Type A insulin resistance without complication |
|  | C10L.00 | Fibrocalculous pancreatopathy |
|  | C10L000 | Fibrocalculous pancreatopathy without complication |
|  | C10M.00 | Lipoatrophic diabetes mellitus |
|  | C10M000 | Lipoatrophic diabetes mellitus without complication |
|  | C10N.00 | Secondary diabetes mellitus |
|  | C10N000 | Secondary diabetes mellitus without complication |
|  | C10N100 | Cystic fibrosis related diabetes mellitus |
|  | C10P.00 | Diabetes mellitus in remission |
|  | C10P000 | Type I diabetes mellitus in remission |
|  | C10P011 | Type 1 diabetes mellitus in remission |
|  | C10P100 | Type II diabetes mellitus in remission |
|  | C10P111 | Type 2 diabetes mellitus in remission |
|  | C10y.00 | Diabetes mellitus with other specified manifestation |
|  | C10y000 | Diabetes mellitus, juvenile, + other specified manifestation |
|  | C10y100 | Diabetes mellitus, adult, + other specified manifestation |
|  | C10yy00 | Other specified diabetes mellitus with other spec comps |
|  | C10yz00 | Diabetes mellitus NOS with other specified manifestation |
|  | C10z.00 | Diabetes mellitus with unspecified complication |
|  | C10z000 | Diabetes mellitus, juvenile type, + unspecified complication |
|  | C10z100 | Diabetes mellitus, adult onset, + unspecified complication |
|  | C10zy00 | Other specified diabetes mellitus with unspecified comps |
|  | C10zz00 | Diabetes mellitus NOS with unspecified complication |
| Hypertension | G2...00 | Hypertensive disease |
|  | G2...11 | BP - hypertensive disease |
|  | G20..00 | Essential hypertension |
|  | G20..11 | High blood pressure |
|  | G20..12 | Primary hypertension |
|  | G200.00 | Malignant essential hypertension |
|  | G201.00 | Benign essential hypertension |
|  | G202.00 | Systolic hypertension |
|  | G203.00 | Diastolic hypertension |
|  | G20z.00 | Essential hypertension NOS |

Table S3 (Continued)

| **Condition** | **Read codes** | **Description** |
| --- | --- | --- |
| Hypertension | G20z.11 | Hypertension NOS |
|  | G21..00 | Hypertensive heart disease |
|  | G210.00 | Malignant hypertensive heart disease |
|  | G210000 | Malignant hypertensive heart disease without CCF |
|  | G210100 | Malignant hypertensive heart disease with CCF |
|  | G210z00 | Malignant hypertensive heart disease NOS |
|  | G211.00 | Benign hypertensive heart disease |
|  | G211000 | Benign hypertensive heart disease without CCF |
|  | G211100 | Benign hypertensive heart disease with CCF |
|  | G211z00 | Benign hypertensive heart disease NOS |
|  | G21z.00 | Hypertensive heart disease NOS |
|  | G21z000 | Hypertensive heart disease NOS without CCF |
|  | G21z011 | Cardiomegaly - hypertensive |
|  | G21z100 | Hypertensive heart disease NOS with CCF |
|  | G21zz00 | Hypertensive heart disease NOS |
|  | G22..00 | Hypertensive renal disease |
|  | G22..11 | Nephrosclerosis |
|  | G220.00 | Malignant hypertensive renal disease |
|  | G221.00 | Benign hypertensive renal disease |
|  | G222.00 | Hypertensive renal disease with renal failure |
|  | G22z.00 | Hypertensive renal disease NOS |
|  | G22z.11 | Renal hypertension |
|  | G23..00 | Hypertensive heart and renal disease |
|  | G230.00 | Malignant hypertensive heart and renal disease |
|  | G231.00 | Benign hypertensive heart and renal disease |
|  | G232.00 | Hypertensive heart&renal dis wth (congestive) heart failure |
|  | G233.00 | Hypertensive heart and renal disease with renal failure |
|  | G234.00 | Hyperten heart&renal dis+both(congestv)heart and renal fail |
|  | G23z.00 | Hypertensive heart and renal disease NOS |
|  | G24..00 | Secondary hypertension |
|  | G240.00 | Secondary malignant hypertension |
|  | G240000 | Secondary malignant renovascular hypertension |
|  | G240z00 | Secondary malignant hypertension NOS |
|  | G241.00 | Secondary benign hypertension |
|  | G241000 | Secondary benign renovascular hypertension |
|  | G241z00 | Secondary benign hypertension NOS |
|  | G244.00 | Hypertension secondary to endocrine disorders |
|  | G24z.00 | Secondary hypertension NOS |
|  | G24z000 | Secondary renovascular hypertension NOS |
|  | G24z100 | Hypertension secondary to drug |
|  | G24zz00 | Secondary hypertension NOS |
|  | G25..00 | Stage 1 hypertension (NICE - Nat Ins for Hth Clin Excl 2011) |

Table S3 (Continued)

| **Condition** | **Read codes** | **Description** |
| --- | --- | --- |
| Hypertension | G25..11 | Stage 1 hypertension |
|  | G250.00 | Stage 1 hyperten (NICE 2011) without evidnce end organ damge |
|  | G251.00 | Stage 1 hyperten (NICE 2011) with evidnce end organ damge |
|  | G26..00 | Severe hypertension (Nat Inst for Health Clinical Ex 2011) |
|  | G26..11 | Severe hypertension |
|  | G27..00 | Hypertension resistant to drug therapy |
|  | G28..00 | Stage 2 hypertension (NICE - Nat Ins for Hth Clin Excl 2011) |
|  | G2y..00 | Other specified hypertensive disease |
|  | G2z..00 | Hypertensive disease NOS |
|  | Gyu2.00 | [X]Hypertensive diseases |
|  | Gyu2000 | [X]Other secondary hypertension |
|  | Gyu2100 | [X]Hypertension secondary to other renal disorders |
| Hypothyroidism | C03..00 | Congenital hypothyroidism |
|  | C03..11 | Cretinism |
|  | C030.00 | Pendred's syndrome |
|  | C031.00 | Goitrous cretin |
|  | C03y.00 | Other specified congenital hypothyroidism |
|  | C03y000 | Congenital hypothyroidism with diffuse goitre |
|  | C03y100 | Congenital hypothyroidism without goitre |
|  | C03z.00 | Congenital hypothyroidism NOS |
|  | C03z.11 | Congenital thyroid insufficiency |
|  | C03z.12 | Cretinism |
|  | C04..00 | Acquired hypothyroidism |
|  | C04..11 | Myxoedema |
|  | C04..12 | Thyroid deficiency |
|  | C04..13 | Hypothyroidism |
|  | C040.00 | Postsurgical hypothyroidism |
|  | C040.11 | Post ablative hypothyroidism |
|  | C041.00 | Other postablative hypothyroidism |
|  | C041000 | Irradiation hypothyroidism |
|  | C041z00 | Postablative hypothyroidism NOS |
|  | C042.00 | Iodine hypothyroidism |
|  | C043.00 | Other iatrogenic hypothyroidism |
|  | C043000 | Hypothyroidism resulting from para-aminosalicylic acid |
|  | C043100 | Hypothyroidism resulting from phenylbutazone |
|  | C043200 | Hypothyroidism resulting from resorcinol |
|  | C043z00 | Iatrogenic hypothyroidism NOS |
|  | C044.00 | Postinfectious hypothyroidism |
|  | C045.00 | Acquired atrophy of thyroid |
|  | C046.00 | Autoimmune myxoedema |
|  | C047.00 | Subclinical hypothyroidism |
|  | C04y.00 | Other acquired hypothyroidism |

Table S3 (Continued)

| **Condition** | **Read codes** | **Description** |
| --- | --- | --- |
| Hypothyroidism | C04z.00 | Hypothyroidism NOS |
|  | C04z.11 | Pretibial myxoedema - hypothyroid |
|  | C04z.12 | Thyroid insufficiency |
|  | C04z.13 | Hypothyroid goitre, acquired |
|  | C04z000 | Premature puberty due to hypothyroidism |
|  | C04z100 | Myxoedema coma |
|  | C0A5.00 | Subclinical iodine-deficiency hypothyroidism |
|  | Cyu1100 | [X]Other specified hypothyroidism |
|  | F381400 | Myasthenic syndrome due to hypothyroidism |
|  | Q433700 | Neonatal jaundice with congenital hypothyroidism |
| Impaired glucose regulation | 44V2.00 | Glucose tolerance. test impaired |
|  | 9mX..00 | Impaired glucose regulation monitoring invitation |
|  | 9mX..00 | Impaired glucose regulation monitoring invitation |
|  | 9mX1.00 | Impaired glucose regulation monitoring invitation 2nd letter |
|  | 9mX1.00 | Impaired glucose regulation monitoring invitation 2nd letter |
|  | 9mX2.00 | Impaired glucose regulation monitoring invitation 3rd letter |
|  | 9mX2.00 | Impaired glucose regulation monitoring invitation 3rd letter |
|  | 9mX3.00 | Impaired glucose regulation monitoring telephone invitation |
|  | 9mX3.00 | Impaired glucose regulation monitoring telephone invitation |
|  | 9mX4.00 | Impaired glucose regulation monitoring verbal invitation |
|  | 9mX4.00 | Impaired glucose regulation monitoring verbal invitation |
|  | 9NS0400 | Referral for impaired glucose tolerance management offered |
|  | C11y200 | Impaired glucose tolerance |
|  | C11y300 | Impaired fasting glycaemia |
|  | C11y400 | Impaired glucose regulation |
|  | C11y500 | Pre-diabetes |
|  | R102.12 | [D]Impaired glucose tolerance test |
|  | R10D000 | [D]Impaired fasting glycaemia |
|  | R10D011 | [D]Impaired fasting glucose |
|  | R10E.00 | [D]Impaired glucose tolerance |
|  | R102.11 | [D]Prediabetes |
|  | C313500 | Glucose intolerance |
