# Supplementary material for: Polycystic ovary syndrome, androgen excess, and the risk of nonalcoholic fatty liver disease in women: A longitudinal study based on a United Kingdom primary care database
Source: PLoS Med. 2018 Mar 28;15(3):e1002542. doi: 10.1371/journal.pmed.1002542 (PMC5873722; doi:10.1371/journal.pmed.1002542)
Supplement: S4 Table — (DOCX) [file pmed.1002542.s006.docx]

S4: Drug codes used to identify antiandrogens, lipid modifying drugs and metformin

| **Drug** | **Drug code** | **Preparation** |
| --- | --- | --- |
| Cyproterone | 85864998 | Co-cyprindiol 2000microgram/35microgram tablets |
|  | 86466998 | Co-cyprindiol 2000microgram/35microgram tablets |
|  | 86925998 | Co-cyprindiol 2mg+35mcg tablets |
|  | 87351998 | Co-cyprindiol 2000microgram/35microgram tablets |
|  | 90826979 | Co-cyprindiol 2000microgram/35microgram tablets |
|  | 90833979 | Co-cyprindiol 2000microgram/35microgram tablets |
|  | 91068998 | Co-cyprindiol 2000microgram/35microgram tablets |
|  | 91069998 | Co-cyprindiol 2000microgram/35microgram tablets |
|  | 94832990 | Co-cyprindiol 2000microgram/35microgram tablets |
|  | 94920998 | Co-cyprindiol 2000microgram/35microgram tablets |
|  | 95220990 | Co-cyprindiol 2000microgram/35microgram tablets |
|  | 95396990 | Co-cyprindiol 2000microgram/35microgram tablets |
|  | 96577998 | Co-cyprindiol 2000microgram/35microgram tablets |
|  | 97520998 | Co-cyprindiol 2000microgram/35microgram tablets |
|  | 81667998 | Cyproterone 100mg tablets |
|  | 81668998 | Cyproterone 50mg tablets |
|  | 92777990 | Cyproterone 50mg tablets |
|  | 96269989 | Cyproterone 100mg tablets |
|  | 96269990 | Cyproterone 50mg tablets |
|  | 96578997 | Cyproterone 100mg tablets |
|  | 96578998 | Cyproterone 50mg tablets |
|  | 97768990 | Cyproterone 50mg tablets |
|  | 98017989 | Cyproterone 100mg tablets |
|  | 98017990 | Cyproterone 50mg tablets |
|  | 98246990 | Cyproterone 50mg tablets |
|  | 99797997 | Cyproterone 100mg tablets |
|  | 99797998 | Cyproterone 50mg tablets |
|  | 99958998 | Cyproterone 50mg tablets |
| Drospirenone | 53008979 | Ethinylestradiol 20microgram / Drospirenone 3mg tablets |
|  | 59254978 | Ethinylestradiol 30microgram / Drospirenone 3mg tablets |
|  | 74455978 | Ethinylestradiol 30microgram / Drospirenone 3mg tablets |
|  | 78546978 | Ethinylestradiol 30microgram / Drospirenone 3mg tablets |
|  | 84583978 | Ethinylestradiol 20microgram / Drospirenone 3mg tablets |
|  | 86831998 | Estradiol 1mg / Drospirenone 2mg tablets |
|  | 86832998 | Estradiol 1mg / Drospirenone 2mg tablets |
|  | 89914979 | Ethinylestradiol 30microgram / Drospirenone 3mg tablets |
|  | 92571998 | Ethinylestradiol 30microgram / Drospirenone 3mg tablets |
|  | 98852998 | Ethinylestradiol 30microgram / Drospirenone 3mg tablets |

Table S4: (Continued)

| **Drug** | **Drug code** | **Preparation** |
| --- | --- | --- |
| other antiandrogen drugs | 82882978 | Enzalutamide 40mg capsules |
|  | 82127998 | Tamsulosin 400microgram / Dutasteride 500microgram capsules |
|  | 82128998 | Tamsulosin 400microgram / Dutasteride 500microgram capsules |
|  | 85997979 | Dutasteride 500microgram capsules |
|  | 89268998 | Dutasteride 500microgram capsules |
|  | 89947998 | Dutasteride 500microgram capsules |
|  | 88336998 | Flutamide 250mg tablets |
|  | 92415979 | Flutamide 250mg tablets |
|  | 93504998 | Flutamide 250mg tablets |
|  | 93505998 | Flutamide 250mg tablets |
|  | 97211990 | Flutamide 250mg tablets |
|  | 97862990 | Flutamide 250mg tablets |
|  | 97867990 | Flutamide 250mg tablets |
|  | 59259978 | Finasteride 1mg tablets |
|  | 63181979 | Finasteride 5mg/5ml oral suspension |
|  | 89063998 | Finasteride 1mg tablets |
|  | 89990979 | Finasteride 1mg tablets |
|  | 91684998 | Finasteride 1mg tablets |
|  | 93144979 | Finasteride 5mg tablets |
|  | 99249998 | Finasteride 5mg tablets |
|  | 99250998 | Finasteride 5mg tablets |
| metformin | 85622998 | Pioglitazone 15mg / Metformin 850mg tablets |
|  | 85625998 | Pioglitazone 15mg / Metformin 850mg tablets |
|  | 85624998 | Pioglitazone 15mg / Metformin 850mg tablets |
|  | 53006979 | Saxagliptin 2.5mg / Metformin 1g tablets |
|  | 76993978 | Dapagliflozin 5mg / Metformin 1g tablets |
|  | 76992978 | Dapagliflozin 5mg / Metformin 1g tablets |
|  | 76991978 | Dapagliflozin 5mg / Metformin 850mg tablets |
|  | 78730978 | Alogliptin 12.5mg / Metformin 1g tablets |
|  | 83401998 | Metformin 1g / Sitagliptin 50mg tablets |
|  | 78729978 | Alogliptin 12.5mg / Metformin 1g tablets |
|  | 84008998 | Vildagliptin 50mg / Metformin 1g tablets |
|  | 84011998 | Vildagliptin 50mg / Metformin 850mg tablets |
|  | 84009998 | Vildagliptin 50mg / Metformin 850mg tablets |
|  | 84010998 | Vildagliptin 50mg / Metformin 1g tablets |
|  | 82068998 | Metformin 1g / Sitagliptin 50mg tablets |
|  | 53004979 | Saxagliptin 2.5mg / Metformin 850mg tablets |
|  | 53005979 | Saxagliptin 2.5mg / Metformin 850mg tablets |
|  | 53007979 | Saxagliptin 2.5mg / Metformin 1g tablets |
|  | 54904979 | Linagliptin 2.5mg / Metformin 850mg tablets |
|  | 54907979 | Linagliptin 2.5mg / Metformin 1g tablets |
|  | 54905979 | Linagliptin 2.5mg / Metformin 850mg tablets |

Table S4: (Continued)

| **Drug** | **Drug code** | **Preparation** |
| --- | --- | --- |
| Metformin | 54906979 | Linagliptin 2.5mg / Metformin 1g tablets |
|  | 82919998 | Metformin 500mg oral powder sachets sugar free |
|  | 82918998 | Metformin 1g oral powder sachets sugar free |
|  | 82917998 | Metformin hydrochloride 500mg sachets |
|  | 82916998 | Metformin hydrochloride 1g sachets |
|  | 81344998 | Metformin 1g modified-release tablets |
|  | 83733998 | Metformin 750mg modified-release tablets |
|  | 83031998 | Metformin 1g modified-release tablets |
|  | 83732998 | Metformin 750mg modified-release tablets |
|  | 79512979 | Metformin 500mg/5ml oral solution |
|  | 83619998 | Metformin 500mg modified-release tablets |
|  | 81158998 | Metformin 500mg modified-release tablets |
|  | 81701998 | Metformin 500mg modified-release tablets |
|  | 83032998 | Metformin 1g modified-release tablets |
|  | 85673998 | Metformin 500mg/5ml oral solution sugar free |
|  | 85674998 | Metformin 500mg/5ml oral solution |
|  | 89129979 | Metformin 1g modified-release tablets |
|  | 89870979 | Metformin 500mg modified-release tablets |
|  | 87053998 | Metformin 500mg modified-release tablets |
|  | 87536998 | Metformin 500mg/5ml oral solution |
|  | 87054998 | Metformin 500mg modified-release tablets |
|  | 89868979 | Metformin 500mg modified-release tablets |
|  | 87883998 | Metformin 500mg modified-release tablets |
|  | 89128979 | Metformin 1g modified-release tablets |
|  | 89188979 | Metformin 500mg/5ml oral solution |
|  | 91221998 | Metformin hydrochloride 500mg tablets |
|  | 93167990 | Metformin 500mg/5ml oral solution |
|  | 91221997 | Metformin hydrochloride 850mg tablets |
|  | 93469979 | Metformin 500mg tablets |
|  | 92983990 | Metformin 500mg/5ml oral solution |
|  | 94235992 | Metformin 500mg tablets |
|  | 94248990 | Metformin 500mg tablets |
|  | 95271992 | Metformin 850mg tablets |
|  | 95270992 | Metformin 800 mg tab |
|  | 96111990 | Metformin 500mg tablets |
|  | 95880998 | Metformin 500mg modified-release tablets |
|  | 96270990 | Metformin 500mg tablets |
|  | 97087998 | Metformin 500mg tablets |
|  | 97110990 | Metformin 500mg tablets |
|  | 97110989 | Metformin 850mg tablets |
|  | 97087997 | Metformin 850mg tablets |
|  | 95272992 | Metformin 250 mg tab |

Table S4: (Continued)

| **Drug** | **Drug code** | **Preparation** |
| --- | --- | --- |
| Metformin | 96850990 | Metformin 500mg tablets |
|  | 95600990 | Metformin 500mg tablets |
|  | 99514990 | Metformin 500mg tablets |
|  | 98125990 | Metformin 500mg tablets |
|  | 99149990 | Metformin 500mg tablets |
|  | 99590997 | Metformin 850mg tablets |
|  | 99514989 | Metformin 850mg tablets |
|  | 99513990 | Metformin 500mg tablets |
|  | 99513989 | Metformin 850mg tablets |
|  | 98125989 | Metformin 850mg tablets |
|  | 98654989 | Metformin 500mg tablets |
|  | 99590998 | Metformin 500mg tablets |
|  | 98493989 | Metformin 500mg tablets |
|  | 98654990 | Metformin 850mg tablets |
|  | 98493990 | Metformin 850mg tablets |
|  | 76199978 | Metformin 1g modified-release tablets |
|  | 71281979 | Metformin 500mg/5ml oral solution |
|  | 54786979 | Metformin 500mg modified-release tablets |
|  | 58558979 | Metformin 500mg modified-release tablets |
|  | 74441978 | Metformin 1g modified-release tablets |
|  | 74454978 | Metformin 500mg modified-release tablets |
|  | 74453978 | Metformin 500mg modified-release tablets |
|  | 87165998 | Metformin with rosiglitazone 1000mg + 4mg tablet |
|  | 87166998 | Metformin with rosiglitazone 1000mg + 2mg tablet |
|  | 87180998 | Metformin & rosiglitazone 1g+2mg tablets |
|  | 87181998 | Rosiglitazone 4mg / metformin 1g tablets |
|  | 87182998 | Rosiglitazone 2mg / metformin 1g tablets |
|  | 87179998 | Metformin & rosiglitazone 1g+4mg tablets |
|  | 87773998 | Rosiglitazone 2mg / metformin 500mg tablets |
|  | 87772998 | Metformin with rosiglitazone 500mg + 2mg tablet |
|  | 87774998 | Metformin with rosiglitazone 500mg + 1mg tablet |
|  | 87771998 | Metformin & rosiglitazone 500mg+1mg tablets |
|  | 87775998 | Rosiglitazone 1mg / metformin 500mg tablets |
|  | 87770998 | Metformin & rosiglitazone 500mg+2mg tablets |
| Lipid modifying drugs | 81048998 | Atorvastatin 20mg chewable tablets sugar free |
|  | 81051998 | Atorvastatin 10mg chewable tablets sugar free |
|  | 83099998 | Simvastatin 40mg/5ml oral suspension sugar free |
|  | 82655998 | Nicotinic acid & laropiprant 1g+20mg tablets |
|  | 83030998 | Simvastatin 80mg tablets |
|  | 81050998 | Atorvastatin 10mg chewable tablets sugar free |
|  | 84268998 | Colesevelam 625mg tablets |
|  | 84267998 | Colesevelam 625mg tablets |

Table S4: (Continued)

| **Drug** | **Drug code** | **Preparation** |
| --- | --- | --- |
| Lipid modifying drugs | 83594998 | Nicotinic acid 1g / laropiprant 20mg modified-release tablets |
|  | 79254979 | Simvastatin 20mg/5ml oral suspension sugar free |
|  | 83188998 | Bezafibrate 200mg tablets |
|  | 83187998 | Bezafibrate 400mg modified-release tablets |
|  | 81049998 | Atorvastatin 20mg chewable tablets sugar free |
|  | 82141978 | Eicosapentaenoic acid 460mg / Docosahexaenoic acid 380mg capsules |
|  | 87853998 | Nicotinic acid 1g modified-release tablets |
|  | 87852998 | Nicotinic acid 500mg modified release tablets |
|  | 89154996 | Cerivastatin 300microgram tablets |
|  | 86791998 | Simvastatin 80mg / Ezetimibe 10mg tablets |
|  | 87854998 | Nicotinic acid 750mg modified-release tablets |
|  | 89153996 | Cerivastatin sodium 300mcg tablets |
|  | 88298997 | Fenofibrate micronised 267mg capsules |
|  | 88534998 | Rosuvastatin 10mg tablets |
|  | 86794998 | Simvastatin 80mg / Ezetimibe 10mg tablets |
|  | 86510979 | Ispaghula husk 3.5g sugar free granules |
|  | 87025998 | Bezafibrate 400mg modified-release tablets |
|  | 87418998 | Simvastatin 10mg tablets |
|  | 87918998 | Simvastatin 10mg tablets |
|  | 89401998 | Bezafibrate 400mg modified-release tablets |
|  | 87917998 | Simvastatin 20mg tablets |
|  | 89089998 | Bezafibrate 400mg modified release tablets |
|  | 87373998 | Simvastatin 10mg tablets |
|  | 87760998 | Colestipol 5g granules sachets sugar free |
|  | 86798998 | Simvastatin 20mg / Ezetimibe 10mg tablets |
|  | 88297996 | Fenofibrate micronised 267mg capsules |
|  | 87848998 | Nicotinic acid pack |
|  | 86796998 | Simvastatin 40mg / Ezetimibe 10mg tablets |
|  | 87849998 | Nicotinic acid 375mg + 500mg + 750mg modified-release tablet |
|  | 87850998 | Nicotinic acid 1g modified release tablets |
|  | 87851998 | Nicotinic acid 750mg modified release tablets |
|  | 86797998 | Simvastatin 20mg / Ezetimibe 10mg tablets |
|  | 87916998 | Simvastatin 40mg tablets |
|  | 89306996 | Atorvastatin 40mg tablets |
|  | 89311998 | Atorvastatin 10mg tablets |
|  | 89617998 | Ispaghula husk 3.5g sugar free granules |
|  | 89154997 | Cerivastatin 200microgram tablets |
|  | 86795998 | Simvastatin 40mg / Ezetimibe 10mg tablets |
|  | 89311997 | Atorvastatin 20mg tablets |
|  | 89306998 | Atorvastatin 10mg tablets |
|  | 89311996 | Atorvastatin 40mg tablets |
|  | 88298996 | Fenofibrate micronised 200mg capsules |

Table S4: (Continued)

| **Drug** | **Drug code** | **Preparation** |
| --- | --- | --- |
| Lipid modifying drugs | 86788998 | Simvastatin 40mg / Ezetimibe 10mg tablets |
|  | 86789998 | Simvastatin 20mg / Ezetimibe 10mg tablets |
|  | 89153998 | Cerivastatin sodium 100mcg tablets |
|  | 86787998 | Simvastatin 80mg / Ezetimibe 10mg tablets |
|  | 89154998 | Cerivastatin 100microgram tablets |
|  | 88297998 | Fenofibrate micronised 67mg capsules |
|  | 86467998 | Rosuvastatin 5mg tablets |
|  | 89285979 | Nicotinic acid 500mg modified release tablets |
|  | 89800998 | Eicosapentaenoic acid 460mg / Docosahexaenoic acid 380mg capsules |
|  | 89284979 | Nicotinic acid 750mg modified release tablets |
|  | 88298998 | Fenofibrate micronised 67mg capsules |
|  | 88297997 | Fenofibrate micronised 200mg capsules |
|  | 89306997 | Atorvastatin 20mg tablets |
|  | 87855998 | Nicotinic acid 500mg modified-release tablets |
|  | 89283979 | Nicotinic acid 1g modified release tablets |
|  | 89153997 | Cerivastatin sodium 200mcg tablets |
|  | 86468998 | Rosuvastatin 5mg tablets |
|  | 92447998 | Cerivastatin sodium 400mcg tablets |
|  | 90973998 | Rosuvastatin 20mg tablets |
|  | 93619998 | Simvastatin 10mg tablets |
|  | 92408998 | Rosuvastatin 20mg tablets |
|  | 93620996 | Simvastatin 40mg tablets |
|  | 92448997 | Cerivastatin 800microgram tablets |
|  | 92410998 | Rosuvastatin 40mg tablets |
|  | 93620997 | Simvastatin 20mg tablets |
|  | 93620998 | Simvastatin 10mg tablets |
|  | 93871990 | Simvastatin 40mg tablets |
|  | 92409998 | Rosuvastatin 10mg tablets |
|  | 93243996 | Pravastatin 40mg tablets |
|  | 91194998 | Fluvastatin 80mg modified-release tablets |
|  | 93010990 | Colestyramine 4g oral powder sachets sugar free |
|  | 92549990 | Fenofibrate micronised 200mg capsules |
|  | 93244998 | Pravastatin 10mg tablets |
|  | 93244997 | Pravastatin 20mg tablets |
|  | 93244996 | Pravastatin 40mg tablets |
|  | 92539998 | Rosuvastatin 40mg tablets |
|  | 90310998 | Atorvastatin 80mg tablets |
|  | 93243997 | Pravastatin 20mg tablets |
|  | 92448998 | Cerivastatin 400microgram tablets |
|  | 94407990 | Simvastatin 20mg tablets |
|  | 93838990 | Bezafibrate 200mg tablets |
|  | 92471998 | Simvastatin 80mg tablets |

Table S4: (Continued)

| **Drug** | **Drug code** | **Preparation** |
| --- | --- | --- |
| Lipid modifying drugs | 93851992 | Colestipol 5g granules sachets sugar free |
|  | 90309998 | Atorvastatin 80mg tablets |
|  | 92460998 | Fenofibrate micronised 160mg tablets |
|  | 91316998 | Colestyramine sugar free powder |
|  | 93243998 | Pravastatin 10mg tablets |
|  | 92292998 | Ezetimibe 10mg tablets |
|  | 92154990 | Simvastatin 20mg/5ml oral suspension sugar free |
|  | 93541998 | Colestyramine 4g oral powder sachets |
|  | 93542998 | Colestyramine 4g oral powder sachets sugar free |
|  | 92804997 | Fluvastatin 40mg capsules |
|  | 94189997 | Fenofibrate micronised 200mg capsules |
|  | 92293998 | Ezetimibe 10mg tablets |
|  | 90649998 | Fenofibrate 200mg capsules |
|  | 92805998 | Fluvastatin 20mg capsules |
|  | 92804998 | Fluvastatin 20mg capsules |
|  | 92805997 | Fluvastatin 40mg capsules |
|  | 93619996 | Simvastatin 40mg tablets |
|  | 92220998 | Simvastatin 80mg tablets |
|  | 90653998 | Colestyramine 4g oral powder sachets sugar free |
|  | 94188997 | Fenofibrate micronised 200mg capsules |
|  | 94112992 | Cholestyramine 325 mg cap |
|  | 94188998 | Fenofibrate 100mg capsule |
|  | 94189998 | Fenofibrate 100mg capsules |
|  | 92804996 | Fluvastatin 80mg modified-release tablets |
|  | 93619997 | Simvastatin 20mg tablets |
|  | 95480990 | Simvastatin 10mg tablets |
|  | 95479990 | Simvastatin 20mg tablets |
|  | 95952997 | Bezafibrate 400mg modified-release tablets |
|  | 95550990 | Simvastatin 20mg tablets |
|  | 95551990 | Simvastatin 10mg tablets |
|  | 94925998 | Eicosapentaenoic acid 170mg / Docosahexaenoic acid 115mg capsules |
|  | 95478990 | Simvastatin 40mg tablets |
|  | 95471990 | Simvastatin 40mg tablets |
|  | 95475990 | Simvastatin 20mg tablets |
|  | 94799998 | Fenofibrate micronised 160mg tablets |
|  | 96295997 | Gemfibrozil 600mg tablets |
|  | 95474990 | Simvastatin 40mg tablets |
|  | 95472990 | Simvastatin 20mg tablets |
|  | 95451990 | Simvastatin 10mg tablets |
|  | 95549990 | Simvastatin 40mg tablets |
|  | 96295998 | Gemfibrozil 300mg capsules |
|  | 94927990 | Simvastatin 80mg tablets |

Table S4: (Continued)

| **Drug** | **Drug code** | **Preparation** |
| --- | --- | --- |
| Lipid modifying drugs | 94827992 | Colestyramine 4g oral powder sachets |
|  | 95501990 | Simvastatin 40mg tablets |
|  | 94782990 | Pravastatin 20mg tablets |
|  | 95185990 | Simvastatin 80mg tablets |
|  | 95494990 | Simvastatin 20mg tablets |
|  | 94605998 | Colestipol 5g granules sachets sugar free |
|  | 95495990 | Simvastatin 10mg tablets |
|  | 94851990 | Pravastatin 10mg tablets |
|  | 95500990 | Simvastatin 80mg tablets |
|  | 94830990 | Pravastatin 20mg tablets |
|  | 95502990 | Simvastatin 20mg tablets |
|  | 97078998 | Fish oil concentrate 1g capsules |
|  | 96685990 | Bezafibrate 400mg modified-release tablets |
|  | 96685989 | Bezafibrate 200mg tablets |
|  | 97377979 | Cerivastatin sodium 300mcg tablets |
|  | 94831990 | Pravastatin 10mg tablets |
|  | 97078997 | Fish oil concentrate oral liquid |
|  | 94661998 | Colestipol 5g granules sachets sugar free |
|  | 95482990 | Simvastatin 20mg tablets |
|  | 95483990 | Simvastatin 10mg tablets |
|  | 95486990 | Simvastatin 40mg tablets |
|  | 95508990 | Simvastatin 10mg tablets |
|  | 95487990 | Simvastatin 20mg tablets |
|  | 95952998 | Bezafibrate 200mg tablets |
|  | 97078996 | Fish oil concentrate oral emulsion |
|  | 94850990 | Pravastatin 20mg tablets |
|  | 94849990 | Pravastatin 40mg tablets |
|  | 94662998 | Colestipol 5g granules sachets sugar free |
|  | 95493990 | Simvastatin 40mg tablets |
|  | 95847990 | Colestyramine 4g oral powder sachets sugar free |
|  | 95098992 | Hexopal 200 mg tab |
|  | 97455979 | Pravastatin 10mg tablets |
|  | 95481990 | Simvastatin 40mg tablets |
|  | 94661997 | Colestipol 5g granules sachets sugar free |
|  | 97430979 | Fluvastatin 20mg capsules |
|  | 95805998 | Bezafibrate 400mg modified release tablets |
|  | 95405990 | Simvastatin 40mg tablets |
|  | 94789990 | Pravastatin 10mg tablets |
|  | 95401998 | Probucol 250mg tablet |
|  | 97247997 | Gemfibrozil 600mg tablets |
|  | 97247998 | Gemfibrozil 300mg capsules |
|  | 96134990 | Colestyramine 4g oral powder sachets |

Table S4: (Continued)

| **Drug** | **Drug code** | **Preparation** |
| --- | --- | --- |
| Lipid modifying drugs | 95278990 | Simvastatin 20mg tablets |
|  | 95277990 | Simvastatin 40mg tablets |
|  | 95372990 | Simvastatin 40mg tablets |
|  | 95406990 | Simvastatin 20mg tablets |
|  | 96642998 | Clofibrate 500mg capsules |
|  | 94925997 | Eicosapentaenoic acid 170mg/g / docosahexaenoic acid 115mg/g oral liquid |
|  | 95450990 | Simvastatin 20mg tablets |
|  | 97433979 | Ciprofibrate 100mg tablets |
|  | 96656990 | Bezafibrate 200mg tablets |
|  | 97454979 | Pravastatin 20mg tablets |
|  | 94883998 | Acipimox 250mg capsules |
|  | 94882998 | Acipimox 250mg capsules |
|  | 95279990 | Simvastatin 10mg tablets |
|  | 95445990 | Simvastatin 10mg tablets |
|  | 96021990 | Fenofibrate micronised 200mg capsules |
|  | 95442990 | Simvastatin 80mg tablets |
|  | 95449990 | Simvastatin 40mg tablets |
|  | 95448990 | Simvastatin 80mg tablets |
|  | 95443990 | Simvastatin 40mg tablets |
|  | 94920990 | Simvastatin 20mg tablets |
|  | 97529979 | Fenofibrate micronised 160mg tablets |
|  | 99957979 | Atorvastatin 80mg tablets |
|  | 98066998 | Nicotinic acid 25mg tablet |
|  | 97518979 | Simvastatin 10mg tablets |
|  | 97551979 | Fenofibrate micronised 200mg capsules |
|  | 97476979 | Simvastatin 40mg tablets |
|  | 97594979 | Bezafibrate 400mg modified-release tablets |
|  | 97723998 | Ciprofibrate 100mg tablets |
|  | 97756979 | Atorvastatin 40mg tablets |
|  | 97495979 | Simvastatin 20mg tablets |
|  | 97655990 | Colestyramine 4g oral powder sachets |
|  | 97751998 | Ciprofibrate 100mg tablets |
|  | 99197998 | Colestyramine 4g oral powder sachets |
|  | 97559979 | Fenofibrate micronised 200mg capsules |
|  | 98455998 | Clofibrate 500mg capsules |
|  | 97482979 | Simvastatin 40mg tablets |
|  | 97895997 | Bezafibrate 400mg modified-release tablets |
|  | 98066996 | Nicotinic acid 100mg tablet |
|  | 97808989 | Gemfibrozil 600mg tablets |
|  | 97595979 | Bezafibrate 400mg modified-release tablets |
|  | 97895998 | Bezafibrate 200mg tablets |
|  | 97800992 | Nicotinic acid 500mg modified-release tablets |

Table S4: (Continued)

| **Drug** | **Drug code** | **Preparation** |
| --- | --- | --- |
| Lipid modifying drugs | 97541979 | Fenofibrate micronised 267mg capsules |
|  | 97494979 | Simvastatin 20mg tablets |
|  | 97508979 | Simvastatin 10mg tablets |
|  | 97612979 | Cerivastatin sodium 200mcg tablets |
|  | 98066997 | Nicotinic acid 50mg tablets |
|  | 99014998 | Bezafibrate 400mg modified-release tablets |
|  | 99456998 | Probucol 250mg tablets |
|  | 97705979 | Cerivastatin sodium 100mcg tablets |
|  | 99725990 | Nicotinic acid 50mg tablets |
|  | 58658979 | Atorvastatin 10mg tablets |
|  | 61212979 | Atorvastatin 20mg chewable tablets sugar free |
|  | 62570979 | Simvastatin 40mg/5ml oral suspension sugar free |
|  | 59480979 | Fluvastatin 20mg capsules |
|  | 57833979 | Atorvastatin 10mg tablets |
|  | 62597979 | Simvastatin 20mg/5ml oral suspension sugar free |
|  | 61213979 | Atorvastatin 20mg chewable tablets sugar free |
|  | 58151979 | Atorvastatin 60mg tablets |
|  | 58671979 | Atorvastatin 40mg tablets |
|  | 64839979 | Simvastatin 40mg/5ml oral suspension sugar free |
|  | 61489979 | Simvastatin 20mg tablets |
|  | 54777979 | Eicosapentaenoic acid 460mg / Docosahexaenoic acid 380mg capsules |
|  | 58668979 | Atorvastatin 40mg tablets |
|  | 58669979 | Atorvastatin 40mg tablets |
|  | 57838979 | Atorvastatin 40mg tablets |
|  | 76814978 | Eicosapentaenoic acid 460mg / Docosahexaenoic acid 380mg capsules |
|  | 72952978 | Eicosapentaenoic acid 460mg / Docosahexaenoic acid 380mg capsules |
|  | 57832979 | Atorvastatin 20mg tablets |
|  | 52277979 | Eicosapentaenoic acid 460mg / Docosahexaenoic acid 380mg capsules |
|  | 58654979 | Atorvastatin 20mg tablets |
|  | 58711979 | Atorvastatin 80mg tablets |
|  | 58653979 | Atorvastatin 20mg tablets |
|  | 61215979 | Atorvastatin 10mg chewable tablets sugar free |
|  | 58661979 | Atorvastatin 10mg tablets |
|  | 86020998 | Simvastatin 20mg/5ml oral solution sugar free |
|  | 92329998 | Eicosapentaenoic acid 460mg / docosahexaenoic acid 380mg capsules |
|  | 58153979 | Atorvastatin 30mg tablets |
|  | 64582979 | Atorvastatin 40mg/5ml oral suspension |
|  | 64584979 | Atorvastatin 40mg/5ml oral solution |
|  | 64586979 | Atorvastatin 10mg/5ml oral suspension |
|  | 64588979 | Atorvastatin 10mg/5ml oral solution |
|  | 64634979 | Atorvastatin 20mg/5ml oral suspension |
|  | 79256979 | Simvastatin 10mg/5ml oral suspension |
