# Supplementary material for: Polycystic ovary syndrome, androgen excess, and the risk of nonalcoholic fatty liver disease in women: A longitudinal study based on a United Kingdom primary care database
Source: PLoS Med. 2018 Mar 28;15(3):e1002542. doi: 10.1371/journal.pmed.1002542 (PMC5873722; doi:10.1371/journal.pmed.1002542)
Supplement: S5 Table — (DOCX) [file pmed.1002542.s007.docx]

S5: Hazard of women with PCOS to develop NAFLD compared to women without PCOS using diabetes censored data

|  | **Diabetes censored data** | | **DM and IGR censored data** | |
| --- | --- | --- | --- | --- |
|  | **PCOS**  **(Exposed)** | **Controls**  **(Unexposed)** | **PCOS**  **(Exposed)** | **Controls**  **(Unexposed)** |
| Total number of participants | 61,841 | 119,373 | 61,557 | 119,077 |
| Person years | 291,198 | 567,775 | 290,103 | 566,580 |
| Incident NAFLD n (%) | 243 (0.39) | 211 (0.18) | 239 (0.39) | 208 (0.17) |
| Incidence rates per 10,000 person years | 8.34 | 3.72 | 8.24 | 3.67 |
| Hazard Ratio (95% CI) | 2.27 (1.89 to 2.73) | | 2.28 (1.89 to 2.75) | |
| p-value | <0.001 | | <0.001 | |
| Adjusted Hazard Ratio (95% CI)* | 2.22 (1.85 to 2.68) | | 2.25 (1.87 to 2.72) | |
| p-value | <0.001 | | <0.001 | |

* Adjusted for age, Townsend score, BMI, diabetes or impaired glucose regulation and hypothyroidism at baseline
