# Supplementary material for: Polycystic ovary syndrome, androgen excess, and the risk of nonalcoholic fatty liver disease in women: A longitudinal study based on a United Kingdom primary care database
Source: PLoS Med. 2018 Mar 28;15(3):e1002542. doi: 10.1371/journal.pmed.1002542 (PMC5873722; doi:10.1371/journal.pmed.1002542)
Supplement: S6 Table — (DOCX) [file pmed.1002542.s008.docx]

S6: Regression model estimates for hazard of women with PCOS to develop NAFLD compared to women without PCOS (n= 184,274)

| **Characteristics** | **Hazard ratio** |  | **P value** |
| --- | --- | --- | --- |
|  |  | **95% CI** |  |
| **PCOS** | 2.23 | (1.86, 2.66) | <0.001 |
| **Age** | 1.05 | (1.03, 1.06) | <0.001 |
|  |  |  |  |
| **Townsend index** |  |  |  |
| 1 | 1.00 |  |  |
| 2 | 1.10 | (0.81, 1.49) | 0.54 |
| 3 | 1.21 | (0.90, 1.61) | 0.20 |
| 4 | 1.47 | (1.11, 1.95) | 0.01 |
| 5 | 1.53 | (1.13, 2.07) | 0.01 |
| Missing or implausible data | 1.52 | (0.99, 2.35) | 0.06 |
|  |  |  |  |
| **BMI (kg/m^2^) Category** |  |  |  |
| <25 | 1.00 |  |  |
| 25-30 | 3.37 | (2.36, 4.83) | <0.001 |
| >30 | 6.98 | (5.07, 9.60) | <0.001 |
| Missing or implausible data | 1.96 | (1.29, 3.00) | <0.001 |
|  |  |  |  |
| **Diabetes or IGR*** | 2.39 | (1.76, 3.25) | <0.001 |
| **Hypothyroidism baseline** | 1.39 | (0.97, 1.98) | 0.07 |

* IGR, impaired glucose regulation (includes impaired fasting glucose (IFG; fasting plasma glucose 6.1-6.9 mmol/L) and impaired glucose tolerance (IGT; plasma glucose 7.8-11.1 mmol/L measured 120min after ingestion of 75g glucose in the oral glucose tolerance test)
