# Supplementary material for: Polycystic ovary syndrome, androgen excess, and the risk of nonalcoholic fatty liver disease in women: A longitudinal study based on a United Kingdom primary care database
Source: PLoS Med. 2018 Mar 28;15(3):e1002542. doi: 10.1371/journal.pmed.1002542 (PMC5873722; doi:10.1371/journal.pmed.1002542)
Supplement: S7 Table — (DOCX) [file pmed.1002542.s009.docx]

S7: Subgroup analysis for hazard of women with PCOS to develop NAFLD compared to women without PCOS stratified by BMI category

|  | **BMI <25kg/m2** | | **25-30Kg/m2** | | **>30Kg/m2** | |
| --- | --- | --- | --- | --- | --- | --- |
|  | **PCOS**  **(Exposed)** | **Controls**  **(Unexposed)** | **PCOS**  **(Exposed)** | **Controls**  **(Unexposed)** | **PCOS**  **(Exposed)** | **Controls**  **(Unexposed)** |
| Total number of participants | 21,689 | 44,742 | 12,412 | 24,656 | 19,499 | 32,627 |
| Person years | 97,957 | 205,355 | 58,842 | 118,646 | 93,157 | 158,056 |
| Incident NAFLD n (%) | 21 (0.10) | 23 (0.05) | 48 (0.39) | 47 (0.19) | 173 (0.89) | 142 (0.44) |
| Incidence rates per 10,000 person years | 2.14 | 1.12 | 8.16 | 4.0 | 18.57 | 8.98 |
| Hazard Ratio (95% CI) | 1.91 (1.06 to 3.45) | | 2.05 (1.37 to 3.07) | | 2.06 (1.65 to 2.57) | |
| p-value | 0.03 | | <0.001 | | <0.001 | |
| Adjusted Hazard Ratio (95% CI)* | 1.85 (1.02 to 3.34) | | 2.05 (1.37 to 3.07) | | 2.06 (1.65 to 2.58) | |
| p-value | 0.043 | | 0.001 | | <0.001 | |

* Adjusted for age, Townsend score, BMI, diabetes or impaired glucose regulation and hypothyroidism at baseline
