# Supplementary material for: Polycystic ovary syndrome, androgen excess, and the risk of nonalcoholic fatty liver disease in women: A longitudinal study based on a United Kingdom primary care database
Source: PLoS Med. 2018 Mar 28;15(3):e1002542. doi: 10.1371/journal.pmed.1002542 (PMC5873722; doi:10.1371/journal.pmed.1002542)
Supplement: S8 Table — (DOCX) [file pmed.1002542.s010.docx]

S8: Regression model estimates for Impact of BMI and PCOS on hazard of women with PCOS to develop NAFLD compared to women without PCOS (n= 184,274)

| **Variable** | **Hazard Ratio** |  | **P value** | |
| --- | --- | --- | --- | --- |
|  |  | **95% CI** |  |  |
| **Age** | 1.05 | (1.04, 1.06) | <0.001 | |
|  |  |  |  | |
| **Townsend index** |  |  |  | |
| 1 | 1.00 |  |  |  |
| 2 | 1.10 | (0.81, 1.49) | 0.547 | |
| 3 | 1.20 | (0.90, 1.61) | 0.207 | |
| 4 | 1.47 | (1.11, 1.96) | 0.007 | |
| 5 | 1.53 | (1.13, 2.07) | 0.006 | |
| Missing or implausible data | 1.53 | (0.99, 2.36) | 0.057 | |
|  |  |  |  | |
| **Diabetes or IGR*** | 2.29 | (1.65, 3.17) | <0.001 | |
| **Hypothyroidism at baseline** | 1.39 | (0.98, 1.99) | 0.069 | |
| **Body mass index (kg/m^2^) and PCOS** |  |  |  | |
| Control women and BMI < 25 | 1.00 |  |  |  |
| Control and BMI 25-30 | 3.27 | (1.98, 5.38) | <0.001 | |
| Control and BMI >30 | 6.82 | (4.38, 10.61) | <0.001 | |
| Control and Missing BMI | 0.93 | (0.44, 1.96) | 0.858 | |
| PCOS women and <25 | 1.92 | (1.06, 3.46) | 0.031 | |
| PCOS women and 25-30 | 6.75 | (4.10, 11.10) | <0.001 | |
| PCOS women and >30 | 14.12 | (9.11, 21.87) | <0.001 | |
| PCOS women and Missing BMI | 5.80 | (3.40, 9.92) | <0.001 | |

* IGR, impaired glucose regulation (includes impaired fasting glucose (IFG; fasting plasma glucose 6.1-6.9 mmol/L) and impaired glucose tolerance (IGT; plasma glucose 7.8-11.1 mmol/L measured 120min after ingestion of 75g glucose in the oral glucose tolerance test)
