# Supplementary material for: Polycystic ovary syndrome, androgen excess, and the risk of nonalcoholic fatty liver disease in women: A longitudinal study based on a United Kingdom primary care database
Source: PLoS Med. 2018 Mar 28;15(3):e1002542. doi: 10.1371/journal.pmed.1002542 (PMC5873722; doi:10.1371/journal.pmed.1002542)
Supplement: S9 Table — (DOCX) [file pmed.1002542.s011.docx]

S9: Regression model estimates for hazard of women with PCOS to develop NAFLD compared to women without PCOS with addition of PCOS diagnostic features as predictor (n= 184,274)

| **Characteristics** | **Hazard ratio** |  | **P value** |
| --- | --- | --- | --- |
|  |  | **95% CI** |  |
| **PCOS** | 2.07 | (1.72, 2.50) | <0.001 |
| **Age** | 1.05 | 1.04, 1.06) | <0.001 |
|  |  |  |  |
| **Townsend index** |  |  |  |
| 1 | 1.0 |  |  |
| 2 | 1.10 | (0.81, 1.49) | 0.551 |
| 3 | 1.20 | (0.90, 1.60) | 0.216 |
| 4 | 1.46 | (1.10, 1.93) | 0.009 |
| 5 | 1.50 | (1.10, 2.03) | 0.010 |
| Missing or implausible data | 1.51 | (0.98, 2.34) | 0.062 |
|  |  |  |  |
| **BMI (kg/m^2^) Category** |  |  |  |
| <25 | 1.0 |  |  |
| 25-30 | 3.35 | (2.34, 4.79) | <0.001 |
| >30 | 6.89 | (5.00, 9.49) | <0.001 |
| Missing or implausible data | 1.97 | (1.29, 3.00) | 0.002 |
|  |  |  |  |
| **Diabetes or IGR*** | 2.24 | (1.61, 3.10) | <0.001 |
| **Hypothyroidism baseline** | 1.38 | (0.96, 1.97) | 0.078 |
|  |  |  |  |
| **Hirsutism** | 1.37 | (1.06, 1.77) | 0.016 |
| **Alopecia** | 1.54 | (1.16, 2.05) | 0.003 |
| **Acne** | 0.99 | (0.76, 1.27) | 0.910 |

* IGR, impaired glucose regulation (includes impaired fasting glucose (IFG; fasting plasma glucose 6.1-6.9 mmol/L) and impaired glucose tolerance (IGT; plasma glucose 7.8-11.1 mmol/L measured 120min after ingestion of 75g glucose in the oral glucose tolerance test)
