# Supplementary material for: Polycystic ovary syndrome, androgen excess, and the risk of nonalcoholic fatty liver disease in women: A longitudinal study based on a United Kingdom primary care database
Source: PLoS Med. 2018 Mar 28;15(3):e1002542. doi: 10.1371/journal.pmed.1002542 (PMC5873722; doi:10.1371/journal.pmed.1002542)
Supplement: S10 Table — (DOCX) [file pmed.1002542.s012.docx]

**S10:Factors associated with NAFLD amongst Non PCOS women (n=121,064).**

| **Covariates** | **Hazard Ratio** |  | **P value** |
| --- | --- | --- | --- |
|  |  | **95% CI** |  |
| **Age** | 1.06 | (1.04, 1.08) | < 0.001 |
|  |  |  |  |
| **Townsend** |  |  |  |
| 1 | 1.00 |  |  |
| 2 | 1.30 | (0.80, 2.12) | 0.29 |
| 3 | 1.74 | (1.11, 2.72) | 0.02 |
| 4 | 1.72 | (1.09, 2.71) | 0.02 |
| 5 | 1.93 | (1.20, 3.11) | 0.01 |
| Missing | 2.07 | (1.03, 4.14) | 0.04 |
|  |  |  |  |
| **BMI (kg/m^2^) category**  <25 | 1.00 |  |  |
| 25-30 | 3.19 | (1.93, 5.26) | < 0.001 |
| >30 | 6.39 | (4.09,9.99) | < 0.001 |
| Missing | 0.94 | (0.44, 1.96) | 0.86 |
|  |  |  |  |
| **Diabetes or IGR*** | 1.35 | (0.57, 3.17) | < 0.001 |
| **Hypothyroidism** | 1.89 | (1.14, 3.13) | 0.01 |
| **Anovulation** | 1.60 | (1.16, 2.20) | 0.004 |
| **Androgen excess feature** |  |  |  |
| **Hirsutism** | 1.74 | (0.64, 4.75) | 0.28 |
| **Acne** | 1.21 | (0.76, 1.91) | 0.42 |
| **Alopecia** | 1.30 | (0.64, 2.64) | 0.46 |
| **Lipid modifying drugs** | 0.75 | (0.31, 1.79) | 0.51 |
| **Metformin** | 1.83 | (0.71, 4.75) | 0.21 |
| **Antiandrogen drug** | 0.75 | (0.39, 1.47) | 0.41 |
