# Supplementary material for: Polycystic ovary syndrome, androgen excess, and the risk of nonalcoholic fatty liver disease in women: A longitudinal study based on a United Kingdom primary care database
Source: PLoS Med. 2018 Mar 28;15(3):e1002542. doi: 10.1371/journal.pmed.1002542 (PMC5873722; doi:10.1371/journal.pmed.1002542)
Supplement: S11 Table — (DOCX) [file pmed.1002542.s013.docx]

S11: Baseline characteristics in cohort of women with available serum testosterone measurement (n=71,061)

| **Characteristics** | **Serum testosterone concentration categories (nmol/L)** | | | | | | |
| --- | --- | --- | --- | --- | --- | --- | --- |
|  | **< 1** | **1 - 1.49** | **1.5 - 1.99** | **2 - 2.49** | **2.5 - 2.99** | **3 - 3.49** | **≥ 3.5** |
| **Population** n (%) | 23,958 (33.7) | 17,663 (24.9) | 13,528 (19.0) | 8,168 (11.5) | 4,061 (5.7) | 1,835 (2.6) | 1,848 (2.6) |
|  |  |  |  |  |  |  |  |
| **Age** mean (SD) | 34.9 (7.8) | 32.4 (7.8) | 31.0 (7.5) | 29.7 (7.4) | 28.4 (6.9) | 27.6 (6.8) | 27.6 (6.9) |
|  |  |  |  |  |  |  |  |
| **Townsend index** n (%) |  |  |  |  |  |  |  |
| 1 | 5,597 (23.4) | 3,934 (22.3) | 2,939 (21.7) | 1,645 (20.1) | 809 (19.9) | 348 (19.0) | 329 (17.8) |
| 2 | 4,693 (19.6) | 3,364 (19.0) | 2,559 (18.9) | 1,498 (18.3) | 718 (17.7) | 324 (17.7) | 296 (16.0) |
| 3 | 4,988 (20.8) | 3,724 (21.1) | 2,801 (20.7) | 1,730 (21.2) | 860 (21.2) | 419 (22.8) | 378 (20.4) |
| 4 | 4,261 (17.8) | 3,332 (18.9) | 2,636 (19.5) | 1,617 (19.8) | 816 (20.1) | 394 (21.5) | 413 (22.4) |
| 5 | 2,775 (11.6) | 2,197 (12.4) | 1,725 (12.8) | 1,135 (13.9) | 619 (15.2) | 246 (13.4) | 327 (17.7) |
| Missing or implausible data | 1,644 (6.9) | 1,112 (6.3) | 868 (6.4) | 543 (6.6) | 239 (5.9) | 104 (5.7) | 105 (5.7) |
|  |  |  |  |  |  |  |  |
| **BMI (kg/m^2^) category** n (%) |  |  |  |  |  |  |  |
| <25 | 11,326 (47.3) | 7,236 (41.0) | 5,138 (38.0) | 2,875 (35.2) | 1,305 (32.1) | 556 (30.3) | 546 (29.6) |
| 25-30 | 5,060 (21.1) | 3,694 (20.9) | 2,743 (20.3) | 1,563 (19.1) | 789 (19.4) | 357 (19.5) | 360 (19.5) |
| >30 | 4,903 (20.5) | 4,282 (24.2) | 3,655 (27.0) | 2,400 (29.4) | 1,259 (31.0) | 601 (32.8) | 628 (34.0) |
| Missing or implausible data | 2,669 (11.1) | 2,451 (13.9) | 1,992 (14.7) | 1,330 (16.3) | 708 (17.4) | 321 (17.5) | 314 (17.0) |

**S11 continued**

| **Characteristics** | **Serum testosterone concentration categories (nmol/L)** | | | | | | |
| --- | --- | --- | --- | --- | --- | --- | --- |
|  | **< 1** | **1 - 1.49** | **1.5 - 1.99** | **2 - 2.49** | **2.5 - 2.99** | **3 - 3.49** | **≥ 3.5** |
| **Smoking status** n (%) |  |  |  |  |  |  |  |
| Smokers | 19,600 (81.8) | 13,409 (75.9) | 9,685 (71.6) | 5,585 (68.4) | 2,725 (67.1) | 1,200 (65.4) | 1,142 (61.8) |
| Smokers | 4,056(16.9) | 3,861 (21.9) | 3,469 (25.6) | 2,288 (28.0) | 1,177 (29.0) | 567 (30.9) | 630 (34.1) |
| Missing | 302 (1.3) | 393 (2.2) | 374 (2.8) | 295 (3.6) | 159 (3.9) | 68 (3.7) | 76 (4.1) |
|  |  |  |  |  |  |  |  |
| **Medical conditions** n (%) |  |  |  |  |  |  |  |
| Diabetes mellitus | 350 (1.5) | 244 (1.4) | 149 (1.1) | 109 (1.3) | 52 (1.3) | 27 (1.5) | 27 (1.5) |
| Hypertension | 648 (2.7) | 394 (2.2) | 330 (2.4) | 187 (2.3) | 95 (2.3) | 44 (2.4) | 46 (2.5) |
| Hypothyroidism | 1,172 (4.9) | 700 (4.0) | 416 (3.1) | 255 (3.1) | 102 (2.5) | 42 (2.3) | 40 (2.2) |
| Impaired glucose regulation | 129 (0.5) | 66 (0.4) | 50 (0.4) | 26 (0.3) | 13 (0.3) | 5 (0.3) | 6 (0.3) |
| PCOS | 419 (1.8) | 426 (2.4) | 371 (2.7) | 260 (3.2) | 159 (3.9) | 82 (4.5) | 76 (4.1) |
