# Supplementary material for: Polycystic ovary syndrome, androgen excess, and the risk of nonalcoholic fatty liver disease in women: A longitudinal study based on a United Kingdom primary care database
Source: PLoS Med. 2018 Mar 28;15(3):e1002542. doi: 10.1371/journal.pmed.1002542 (PMC5873722; doi:10.1371/journal.pmed.1002542)
Supplement: S12 Table — (DOCX) [file pmed.1002542.s014.docx]

S12: Factors associated with NAFLD amongst the cohort of women with available serum testosterone measurement (n=71,061)

| **Covariates** | **Hazard ratio** | **95% CI** | **P Value** |
| --- | --- | --- | --- |
|  |  |  |  |
| **Serum testosterone concentration (nmol/L)** |  |  |  |
| < 1.0 | 1.0 |  |  |
| 1.0 - 1.49 | 1.39 | (0.93, 2.07) | 0.112 |
| 1.5 - 1.99 | 1.04 | (0.66, 1.65) | 0.856 |
| 2.0 - 2.49 | 0.89 | (0.52, 1.55) | 0.684 |
| 2.5 - 2.99 | 1.18 | (0.61, 2.27 | 0.616 |
| 3.0 - 3.49 | 2.30 | (1.16, 4.53) | 0.017 |
| >3.5 | 2.40 | (1.24, 4.66) | 0.009 |
|  |  |  |  |
| **Age** | 1.04 | (1.02, 1.06) | <0.001 |
|  |  |  |  |
| **Townsend index** |  |  |  |
| 1 | 1.00 |  |  |
| 2 | 1.44 | (0.91, 2.28) | 0.124 |
| 3 | 1.07 | (0.66, 1.73) | 0.793 |
| 4 | 1.62 | (1.03, 2.54) | 0.037 |
| 5 | 1.30 | (0.77, 2.19) | 0.328 |
| Missing or implausible data | 1.55 | (0.74, 3.25) | 0.247 |
|  |  |  |  |
| **BMI category (kg/m^2^)** |  |  |  |
| <25 | 1.00 |  |  |
| 25-30 | 4.47 | (2.45, 8.16) | <0.001 |
| >30 | 10.86 | (6.30, 18.72) | <0.001 |
| Missing or implausible data | 3.68 | (1.85, 7.33) | <0.001 |
| **Hypothyroidism** | 1.20 | (0.66, 2.16) | 0.547 |
| **Diabetes or IGR*** | 2.64 | (1.53, 4.53) | <0.001 |

* IGR, impaired glucose regulation (includes impaired fasting glucose (IFG; fasting plasma glucose 6.1-6.9 mmol/L) and impaired glucose tolerance (IGT; plasma glucose 7.8-11.1 mmol/L measured 120min after ingestion of 75g glucose in the oral glucose tolerance
