# Supplementary material for: Polycystic ovary syndrome, androgen excess, and the risk of nonalcoholic fatty liver disease in women: A longitudinal study based on a United Kingdom primary care database
Source: PLoS Med. 2018 Mar 28;15(3):e1002542. doi: 10.1371/journal.pmed.1002542 (PMC5873722; doi:10.1371/journal.pmed.1002542)
Supplement: S13 Table — (DOCX) [file pmed.1002542.s015.docx]

**S13: Hazard of women with serum testosterone level ≥ 1 nmol/L to develop NAFLD compared to women with serum testosterone level <1 nmol/L (n=71,061)**

|  | **Serum testosterone concentration categories (nmol/L)** | | | | | | |
| --- | --- | --- | --- | --- | --- | --- | --- |
|  | **< 1** | **1 - 1.49** | **1.5 - 1.99** | **2 - 2.49** | **2.5 - 2.99** | **3 - 3.49** | **≥ 3.5** |
| Number of participants | 23,958 | 17,663 | 13,528 | 8,168 | 4,061 | 1,835 | 1,848 |
| Incident NAFLD n (%) | 44 (0.18) | 53 (0.3) | 34 (0.25) | 19 (0.23) | 12 (0.30) | 11 (0.60) | 12 (0.65) |
| Person years | 85,541 | 73,870 | 62,685 | 39,970 | 19,924 | 9,187 | 9,306 |
| Incidence rate per 10,000 person years | 5.14 | 7.17 | 5.42 | 4.75 | 6.02 | 11.97 | 12.89 |
| Hazard Ratio | 1 | 1.37 | 1.03 | 0.9 | 1.15 | 2.28 | 2.45 |
| 95% CI of Hazard ratio |  | (0.92, 2.05) | (0.66, 1.62) | 0.53, 1.55) | (0.61, 2.18) | (1.17, 4.41) | (1.29,4.64) |
| p-value |  | 0.12 | 0.89 | 0.71 | 0.67 | 0.015 | 0.006 |
| Adjusted Hazard Ratio* | 1 | 1.39 | 1.04 | 0.89 | 1.18 | 2.3 | 2.4 |
| 95% CI Baseline adjusted Hazard Ratio* |  | (0.93, 2.07) | (0.66, 1.65) | (0.52, 1.55) | (0.61, 2.28) | (1.16, 4.53) | (1.24, 4.66) |
| p-value |  | 0.11 | 0.86 | 0.68 | 0.62 | 0.017 | 0.009 |

* Adjusted for age, Townsend score, BMI, diabetes or impaired glucose regulation and hypothyroidism at baseline
