# Supplementary material for: Polycystic ovary syndrome, androgen excess, and the risk of nonalcoholic fatty liver disease in women: A longitudinal study based on a United Kingdom primary care database
Source: PLoS Med. 2018 Mar 28;15(3):e1002542. doi: 10.1371/journal.pmed.1002542 (PMC5873722; doi:10.1371/journal.pmed.1002542)
Supplement: S15 Table — (DOCX) [file pmed.1002542.s017.docx]

S15: Baseline characteristics in the cohort of women with available serum SHBG measurement (n=49,625)

| **Characteristics** |  | | **Serum SHBG concentration categories (nmol/L)** | | | | |
| --- | --- | --- | --- | --- | --- | --- | --- |
|  | **<20** | **20 - 29.99** | | **30 - 39.99** | **40 - 49.99** | **50 - 59.99** | **≥60** |
| **Population** n (%) | 4,544 (9.2) | 7,573 (15.3) | | 7,929 (16.0) | 7,133 (14.4) | 5,796 (11.7) | 16,650 (33.6) |
|  |  |  | |  |  |  |  |
| **Age** mean (SD) | 28.5 (7.5) | 29.8 (7.6) | | 30.6 (7.7) | 31.1 (7.7) | 31.8 (7.6) | 32.4 (7.5) |
|  |  |  | |  |  |  |  |
| **Townsend index** n (%) | |  | |  |  |  |  |
| 1 | 783 (17.2) | 1,363 (18.0) | | 1,527 (19.3) | 1,472 (20.6) | 1,234 (21.3) | 3,555 (21.4) |
| 2 | 718 (15.8) | 1,191 (15.8) | | 1,395 (17.6) | 1,250 (17.5) | 1,022 (17.6) | 3,193 (19.2) |
| 3 | 950 (20.9) | 1,589 (21.0) | | 1,644 (20.7) | 1,461 (20.5) | 1,219 (21.0) | 3,426 (20.6) |
| 4 | 1,041 (22.9) | 1,621 (21.4) | | 1,641 (20.7) | 1,399 (19.6) | 1,139 (19.6) | 3,135 (18.8) |
| 5 | 741 (16.3) | 1,247 (16.5) | | 1,154 (14.6) | 1,036 (14.5) | 749 (12.9) | 1,970 (11.8) |
| Missing or implausible data | 311 (6.8) | 562 (7.4) | | 568 (7.2) | 515 (7.2) | 433 (7.5) | 1,371 (8.2) |
|  |  |  | |  |  |  |  |
| **BMI (kg/m^2^) categorised** n (%) | |  | |  |  |  |  |
| <25 | 509 (11.2) | 1,461 (19.3) | | 2,307 (29.0) | 2,908 (40.8) | 2,809 (48.5) | 9,896 (59.4) |
| 25-30 | 802 (17.6) | 1,568 (20.7) | | 1,836 (23.2) | 1,597 (22.4) | 1,238 (21.4) | 2,882 (17.3) |
| >30 | 2,578 (56.7) | 3,463 (45.7) | | 2,673 (33.7) | 1,591 (22.3) | 924 (15.9) | 1,642 (9.9) |
| Missing or implausible data | 655 (14.4) | 1,081 (14.3) | | 1,113 (14.0) | 1,037 (14.5) | 825 (14.2) | 2,230 (13.4) |
|  |  |  | |  |  |  |  |
| **Smoking status** n (%) |  |  | |  |  |  |  |
| Non-smokers | 3,376 (74.3) | 5,584 (73.7) | | 5,908 (74.5) | 5,379 (75.4) | 4,406 (76.02) | 13,078 (78.6) |
| Smokers | 1,032 (22.7) | 1,799 (23.8) | | 1,850 (23.3) | 1,625 (22.8) | 1,274 (22.0) | 3,344 (20.1) |
| Missing or implausible data | 136 (3.0) | 190 (2.5) | | 171 (2.2) | 129 (1.8) | 116 (2.0) | 228 (1.4) |

**S15 continued**

| **Characteristics** |  | **Serum SHBG concentration categories (nmol/L)** | | | | | |
| --- | --- | --- | --- | --- | --- | --- | --- |
|  | **<20** | | **20 - 29.99** | **30 - 39.99** | **40 - 49.99** | **50 - 59.99** | **≥60** |
| **Medical conditions at baseline** n (%) |  | |  |  |  |  |  |
| Diabetes mellitus | 167 (3.7) | | 123 (1.6) | 92 (1.2) | 79 (1.1) | 48 (0.8) | 127 (0.8) |
| Hypertension | 190 (4.2) | | 255 (3.4) | 182 (2.3) | 138 (1.9) | 107 (1.8) | 211 (1.3) |
| Hypothyroidism | 232 (5.1) | | 313 (4.1) | 298 (3.8) | 283 (4.0) | 213 (3.7) | 608 (3.7) |
| Impaired glucose regulation | 54 (1.2) | | 58 (0.8) | 39 (0.5) | 15 (0.2) | 17 (0.3) | 37 (0.2) |
| PCOS | 441 (9.7) | | 559 (7.4) | 404 (5.1) | 283 (4.0) | 190 (3.3) | 478 (2.9) |
