# Supplementary material for: Polycystic ovary syndrome, androgen excess, and the risk of nonalcoholic fatty liver disease in women: A longitudinal study based on a United Kingdom primary care database
Source: PLoS Med. 2018 Mar 28;15(3):e1002542. doi: 10.1371/journal.pmed.1002542 (PMC5873722; doi:10.1371/journal.pmed.1002542)
Supplement: S16 Table — (DOCX) [file pmed.1002542.s018.docx]

S16: Factors associated with NAFLD amongst the cohort of women with available serum SHBG measurement (n=49,625)

| **Covariate** | **Hazard ratio** |  | **P Value** |
| --- | --- | --- | --- |
|  |  | **95% CI** |  |
| SHBG (nmol/L) category |  |  |  |
| <20 | 4.98 | (2.45, 10.11) | <0.001 |
| 20 - 29.99 | 4.75 | (2.44, 9.25) | <0.001 |
| 30 - 39.99 | 1.66 | (0.77, 3.57) | 0.199 |
| 40 - 49.99 | 2.06 | (0.96, 4.44) | 0.065 |
| 50 - 59.99 | 0.94 | (0.33, 2.67) | 0.903 |
| ≥ 60 | 1.00 |  |  |
|  |  |  |  |
| Age | 1.07 | (1.05, 1.10) | <0.001 |
|  |  |  |  |
| Townsend score # |  |  |  |
| 1 | 1.00 |  |  |
| 2 | 2.31 | (1.24, 4.31) | 0.009 |
| 3 | 1.78 | (0.94, 3.36) | 0.076 |
| 4 | 2.53 | (1.37, 4.69) | 0.003 |
| 5 | 1.84 | (0.92, 3.67) | 0.082 |
| Missing or implausible data | 1.50 | (0.54, 4.15) | 0.431 |
|  |  |  |  |
| BMI (kg/m^2^) Category |  |  |  |
| <25 | 1.00 |  |  |
| 25-30 | 4.08 | (1.97, 8.43) | <0.001 |
| >30 | 5.41 | (2.69, 10.88) | <0.001 |
| Missing or implausible data | 1.87 | (0.73, 4.79) | 0.186 |
|  |  |  |  |
| Hypothyroidism | 1.08 | (0.53, 2.23) | 0.856 |
| Diabetes or IGR* | 1.39 | (0.67, 2.92) | 0.430 |

# Townsend score – presented as quintiles with 1 least deprived and 5 most deprived

* IGR, impaired glucose regulation (includes impaired fasting glucose (IFG; fasting plasma glucose 6.1-6.9 mmol/L) and impaired glucose tolerance (IGT; plasma glucose 7.8-11.1 mmol/L measured 120min after ingestion of 75g glucose in the oral glucose tolerance test)
