# Supplementary material for: Polycystic ovary syndrome, androgen excess, and the risk of nonalcoholic fatty liver disease in women: A longitudinal study based on a United Kingdom primary care database
Source: PLoS Med. 2018 Mar 28;15(3):e1002542. doi: 10.1371/journal.pmed.1002542 (PMC5873722; doi:10.1371/journal.pmed.1002542)
Supplement: S17 Table — (DOCX) [file pmed.1002542.s019.docx]

S17: Hazard of women with serum SHBG level <60 nmol/L to develop NAFLD compared to women with serum SHBG level ≥60 nmol/L (n=49,625)

|  | **Serum SHBG concentration categories (nmol/L)** | | | | | |
| --- | --- | --- | --- | --- | --- | --- |
|  | **<20** | **20 - 29.99** | **30 - 39.99** | **40 - 49.99** | **50 - 59.99** | **≥60** |
| Number of participants | 4,544 | 7,573 | 7,929 | 7,133 | 5,796 | 16,650 |
| Incident NAFLD n (%) | 31 (0.68) | 47 (0.62) | 16 (0.20) | 15 (0.21) | 5 (0.09) | 12 (0.07) |
| Person years | 17,263 | 28,470 | 29,428 | 26,351 | 20,233 | 55,353 |
| Incidence rate per 10,000 years | 17.96 | 16.51 | 5.44 | 5.70 | 2.47 | 2.17 |
| Hazard Ratio | 6.39 | 5.77 | 2.18 | 2.22 | 0.77 | 1 |
| 95% CI of Hazard ratio | (3.64, 11.23) | (3.38, 9.83) | (1.16, 4.09) | (1.16, 4.22) | (0.29, 2.08) |  |
| p-value | <0.001 | <0.001 | 0.015 | 0.016 | 0.61 |  |
| Adjusted Hazard Ratio* | 4.98 | 4.75 | 1.66 | 2.06 | 0.94 | 1 |
| 95% CI Adjusted Hazard Ratio* | (2.45, 10.11) | (2.44, 925) | (0.77, 3.57) | (0.96, 4.44) | (0.33, 2.67) |  |
| p-value | <0.001 | <0.001 | 0.19 | 0.06 | 0.90 |  |

* Adjusted for age, Townsend score, BMI, diabetes or impaired glucose regulation and hypothyroidism at baseline
