# Supplementary material for: Polycystic ovary syndrome, androgen excess, and the risk of nonalcoholic fatty liver disease in women: A longitudinal study based on a United Kingdom primary care database
Source: PLoS Med. 2018 Mar 28;15(3):e1002542. doi: 10.1371/journal.pmed.1002542 (PMC5873722; doi:10.1371/journal.pmed.1002542)
Supplement: S18 Table — (DOCX) [file pmed.1002542.s020.docx]

S18:Risk of NAFLD based on available serum SHBG measurement following exclusion of participants with PCOS/PCO codes (n= 38,784)

| **Covariate** | **Hazard ratio** |  | **P Value** |
| --- | --- | --- | --- |
|  |  | **95% CI** |  |
| SHBG (nmol/L) category |  |  |  |
| <20 | 5.42 | (2.32, 12.64) | <0.001 |
| 20 - 29.99 | 4.85 | (2.22, 10.58) | <0.001 |
| 30 - 39.99 | 1.76 | (0.73, 4.26) | 0.210 |
| 40 - 49.99 | 1.71 | (0.67, 4.35) | 0.259 |
| 50 - 59.99 | 0.98 | (0.30, 2.21) | 0.977 |
| ≥ 60 | 1.00 |  |  |
|  |  |  |  |

*Patients without a known diagnosis of PCOS who had serum SHBG measurements. A limitation of these data is that many of these women with serum SHBG measurements may have had suspected androgen excess, and hence PCOS, despite the fact that this was not coded.
