# Supplementary material for: Polycystic ovary syndrome, androgen excess, and the risk of nonalcoholic fatty liver disease in women: A longitudinal study based on a United Kingdom primary care database
Source: PLoS Med. 2018 Mar 28;15(3):e1002542. doi: 10.1371/journal.pmed.1002542 (PMC5873722; doi:10.1371/journal.pmed.1002542)
Supplement: S1 Text — (DOCX) [file pmed.1002542.s021.docx]

Polycystic ovary syndrome and risk of Non Alcoholic Fatty Liver Disease

# Background

Polycystic ovary syndrome is an under-recognised, common and complex endocrine condition (Teede, Gibson-Helm, Norman, & Boyle, 2014) affecting females of reproductive age. The currently adopted diagnostic criteria for PCOS are the Rotterdam 2003 criteria, which require any two of the three criteria to confirm PCOS namely oligo-ovulation and or anovulation, clinical and or biochemical signs of hyperandrogenism and polycystic ovaries, provided secondary causes are excluded (Legro, 2015).

Main clinical manifestations of PCOS are related to hyperandrogenism or /and anovulation. Hyperannrogenism manifests mainly as hirsutism, with acne and alopecia being common associations (Shivaprakash et al., 2013).

There are many long term complications of PCOS such as an increased risk of developing gestational diabetes, type II diabetes, sleep apnoea, cardiovascular disease, endometrial cancer (Barry, Azizia, & Hardiman, 2014).

The prevalence of PCOS among women of reproductive age (15 to 45 years) in many countries vary from 6 to 8% (Diamanti-Kandarakis et al., 1999) (Michelmore, Balen, Dunger, & Vessey, 1999) (Kumarapeli, Seneviratne, Wijeyaratne, Yapa, & Dodampahala, 2008) (Jiao et al., 2014). Therefore, PCOS is considered the most common endocrine abnormality among young women (Livadas & Diamanti-Kandarakis, 2013).

The healthcare related economic burden of PCOS during reproductive life span in the United States in 2005 was estimated to be 4.36 billion US dollars which arose from initial evaluation (2%), hormonal treatment of menstrual dysfunction or abnormal uterine bleeding (31%), providing infertility care (12.1%), PCOS associated diabetes (40.5%) and in treating hirsutism (14.2%) (Azziz, Marin, Hoq, Badamgarav, & Song, 2005). This analysis has not taken into account the potential for premature cardiovascular outcomes, particularly in more vulnerable ethnic groups and regions of the world.

Although prevalence of PCOS has been widely reported, we could not access literature on the incidence of PCOS.

# Purpose

The purpose of this doctoral research study is to determine the association between PCOS and Non Alcoholic Fatty Liver Disease (NAFLD).

# Methods

# Data source

This study will use THIN data. This retrospective study intends to use general practice data as this is one of the most common longitudinal data available in the UK. Although general practice data is available through many sources such as GPRD and THIN, we chose THIN data base as the University of Birmingham has the access to THIN database. Many of the variables included in this study are part of Quality and Outcomes Framework (QoF), quality of maintenance of this data is closely monitored.

The Health Improvement Network (THIN) database, an electronic primary care data base containing anonymised patient data will be used as the data source in this study. THIN database covers 5.7% of the UK population (Dhalwani, Tata, Coleman, Fleming, & Szatkowski, 2013) and has been proven to be generalizable to UK demographics (Blak, Thompson, Dattani, & Bourke, 2011). More than 800 research papers have been published using the General Practise Research Database which is similar to THIN (Williams, van Staa, Puri, & Eaton, 2012). This database includes approximately two million reproductive aged women (aged 15 to 49 years) and 9.5 million patients from 495 practices in 2013 (Dhalwani et al., 2013). There were around 53 000 prevalent cases and 21 000 incident cases of PCOS in 2012 (Morgan, Jenkins-Jones, Currie, & Rees, 2012). Information available in this dataset includes demography, clinical assessment (diagnoses and health contacts), investigations and prescriptions.

# Analysis

Cohorts of exposed and non-exposed groups will be developed. The inclusion criteria for the exposed group will include PCOS patients diagnosed after one year wash in period from the registration at the practice and not being diagnosed with NAFLD at the time of diagnosis of PCOS. The index date for this group will be the date of diagnosis of PCOS and the exit date will be the date of diagnosis of NAFLD or termination of study period or transfer to another practice or death of the patient and whichever the earliest.

For prevalent PCOS patients (diagnosed before study entry) the date of registration at the practice or when the practice becomes eligible to take part will be considered as the index date for this group.

The criteria for selection of non-exposed group for the comparison with the exposed group with incident cases will include 1) those who do not fulfil the criteria to diagnose PCOS, 2) having one year wash in period prior to the index date of the exposed participant 3) not being diagnosed with NAFLD at the time of diagnosis of PCOS 4) randomly chosen to match with the age (same or one year around the year of birth) and clinical practice of the exposed.

The proportion of exposed to non-exposed will be maintained as 1:4 in each cohort. Comparison will be made in the demographic characteristics between those selected and non-selected among the eligible non-exposed registrants in the data set to assess the randomness of selection of non-exposed groups.

Within the POCS cohort we will explore what factors alter their risk of NAFLD, For example androgen excess features, BMI and antiandrogen medications.

**Endpoints**

The endpoints will be whichever occur first among any of the following 1) diagnosis of NAFLD, 2) patient leaving the practice 3) death of the patient and 4) the termination of documentation in the THIN database.

# Confounding variables

Confounders were chosen through review of literature and controlled using multiple regression analysis and include Age, BMI, smoking, Townsend index for social deprivation, diabetes mellitus, hypothyroidism and impaired glucose regulation (Loomis et al. 2016).

# Reference list

Abdul Sultan, A., Tata, L. J., Grainge, M. J., & West, J. (2013). The incidence of first venous thromboembolism in and around pregnancy using linked primary and secondary care data: a population based cohort study from England and comparative meta-analysis. *PloS One*, *8*(7), e70310. https://doi.org/10.1371/journal.pone.0070310

Azziz, R., Marin, C., Hoq, L., Badamgarav, E., & Song, P. (2005). Health care-related economic burden of the polycystic ovary syndrome during the reproductive life span. *The Journal of Clinical Endocrinology and Metabolism*, *90*(8), 4650–8. https://doi.org/10.1210/jc.2005-0628

Barry, J. A., Azizia, M. M., & Hardiman, P. J. (2014). Risk of endometrial, ovarian and breast cancer in women with polycystic ovary syndrome: a systematic review and meta-analysis. *Human Reproduction Update*, *20*(5), 748–758. https://doi.org/10.1093/humupd/dmu012

Blak, B. T., Thompson, M., Dattani, H., & Bourke, A. (2011). Generalisability of The Health Improvement Network (THIN) database: demographics, chronic disease prevalence and mortality rates. *Informatics in Primary Care*, *19*(4), 251–5. Retrieved from http://www.ncbi.nlm.nih.gov/pubmed/22828580

Dhalwani, N. N., Tata, L. J., Coleman, T., Fleming, K. M., & Szatkowski, L. (2013). Completeness of maternal smoking status recording during pregnancy in United Kingdom primary care data. *PloS One*, *8*(9), e72218. https://doi.org/10.1371/journal.pone.0072218

Diamanti-Kandarakis, E., Kouli, C. R., Bergiele, A. T., Filandra, F. A., Tsianateli, T. C., Spina, G. G., … Bartzis, M. I. (1999). A survey of the polycystic ovary syndrome in the Greek island of Lesbos: hormonal and metabolic profile. *The Journal of Clinical Endocrinology and Metabolism*, *84*(11), 4006–11. https://doi.org/10.1210/jcem.84.11.6148

Jiao, J., Fang, Y., Wang, T., Wang, Z., Zhou, M., & Wang, X. (2014). Epidemiologic investigation of polycystic ovarian syndrome (PCOS) in Han ethnic women of reproductive age in Liaoning Province, China. *Clinical and Experimental Obstetrics & Gynecology*, *41*(3), 304–9. Retrieved from http://www.ncbi.nlm.nih.gov/pubmed/24992782

Kumarapeli, V., Seneviratne, R. D. a, Wijeyaratne, C. N., Yapa, R. M. S. C., & Dodampahala, S. H. (2008). A simple screening approach for assessing community prevalence and phenotype of polycystic ovary syndrome in a semiurban population in Sri Lanka. *American Journal of Epidemiology*, *168*(3), 321–328. https://doi.org/10.1093/aje/kwn137

Legro, R. (2015). Diagnosis and treatment of polycystic ovary syndrome (PCOS): an interview with Richard Legro. *BMC Medicine*, *13*, 64. https://doi.org/10.1186/s12916-015-0299-2

Livadas, S., & Diamanti-Kandarakis, E. (2013). Polycystic ovary syndrome: Definitions, phenotypes and diagnostic approach. *Frontiers of Hormone Research*, *40*, 1–21. https://doi.org/10.1159/000341673

Lui, K. J. (1988). Estimation of sample sizes in case-control studies with multiple controls per case: dichotomous data. *American Journal of Epidemiology*, *127*(5), 1064–70. Retrieved from http://www.ncbi.nlm.nih.gov/pubmed/3358407

Michelmore, K. F., Balen, A. H., Dunger, D. B., & Vessey, M. P. (1999). Polycystic ovaries and associated clinical and biochemical features in young women. *Clinical Endocrinology*, *51*(6), 779–86. Retrieved from http://www.ncbi.nlm.nih.gov/pubmed/10619984

Morgan, C. L., Jenkins-Jones, S., Currie, C. J., & Rees, D. A. (2012). Evaluation of adverse outcome in young women with polycystic ovary syndrome versus matched, reference controls: a retrospective, observational study. *The Journal of Clinical Endocrinology and Metabolism*, *97*(9), 3251–60. https://doi.org/10.1210/jc.2012-1690

Shivaprakash, G., A, B., Kamath, A., Shivaprakash, P., Adhikari, P., Up, R., … Padubidri, J. R. (2013). Acanthosis Nigricansin PCOS Patients and Its Relation with Type 2 Diabetes Mellitus and Body Mass at a Tertiary Care Hospital in Southern India. *Journal of Clinical and Diagnostic Research : JCDR*, *7*(2), 317–9. https://doi.org/10.7860/JCDR/2013/4930.2756

Teede, H., Gibson-Helm, M., Norman, R. J., & Boyle, J. (2014). Polycystic ovary syndrome: perceptions and attitudes of women and primary health care physicians on features of PCOS and renaming the syndrome. *The Journal of Clinical Endocrinology and Metabolism*, *99*(1), E107-11. https://doi.org/10.1210/jc.2013-2978

Williams, T., van Staa, T., Puri, S., & Eaton, S. (2012). Recent advances in the utility and use of the General Practice Research Database as an example of a UK Primary Care Data resource. *Therapeutic Advances in Drug Safety*, *3*(2), 89–99. https://doi.org/10.1177/2042098611435911
